# Branched-chain amino acid catabolism is a conserved regulator of physiological ageing

**DOI:** 10.1038/ncomms10043

**Published:** 2015-12-01

**Authors:** Johannes Mansfeld, Nadine Urban, Steffen Priebe, Marco Groth, Christiane Frahm, Nils Hartmann, Juliane Gebauer, Meenakshi Ravichandran, Anne Dommaschk, Sebastian Schmeisser, Doreen Kuhlow, Shamci Monajembashi, Sibylle Bremer-Streck, Peter Hemmerich, Michael Kiehntopf, Nicola Zamboni, Christoph Englert, Reinhard Guthke, Christoph Kaleta, Matthias Platzer, Jürgen Sühnel, Otto W. Witte, Kim Zarse, Michael Ristow

**Affiliations:** 1 Energy Metabolism Laboratory, Swiss Federal Institute of Technology (ETH) Zurich, CH-8603 Zurich, Switzerland; 2 DFG Graduate School of Adaptive Stress Response #1715, D-07745 Jena, Germany; 3 Department of Human Nutrition, Institute of Nutrition, Friedrich-Schiller-University Jena, D-07743 Jena, Germany; 4 GerontoSysJenAge Consortium, BMBF 0315581, D-07745 Jena, Germany; 5 Biocomputing Group, Leibniz Institute on Aging—Fritz Lipmann Institute, D-07745 Jena, Germany; 6 Systems Biology and Bioinformatics Group, Leibniz Institute for Natural Product Research and Infection Biology, Hans-Knöll-Institute, D-07745 Jena, Germany; 7 Genome Analysis, Leibniz Institute on Aging—Fritz Lipmann Institute, D-07745 Jena, Germany; 8 Hans Berger Department of Neurology, Jena University Hospital, D-07747 Jena, Germany; 9 Molecular Genetics, Leibniz Institute on Aging—Fritz Lipmann Institute, D-07745 Jena, Germany; 10 Research Group Theoretical Systems Biology, Friedrich-Schiller-University Jena, D-07743 Jena, Germany; 11 German Institute of Human Nutrition Potsdam-Rehbrücke, D-14558 Nuthetal, Germany; 12 Imaging Facility, Leibniz Institute on Aging—Fritz Lipmann Institute, D-07745 Jena, Germany; 13 Institute of Clinical Chemistry and Laboratory Medicine, University of Jena, D-07743 Jena, Germany; 14 Institute of Molecular Systems Biology, Swiss Federal Institute of Technology (ETH) Zurich, CH-8093 Zürich, Switzerland; 15 Faculty of Biology and Pharmacy, Friedrich-Schiller-University Jena, 07743 Jena, Germany

## Abstract

Ageing has been defined as a global decline in physiological function depending on both environmental and genetic factors. Here we identify gene transcripts that are similarly regulated during physiological ageing in nematodes, zebrafish and mice. We observe the strongest extension of lifespan when impairing expression of the branched-chain amino acid transferase-1 (*bcat-1*) gene in *C. elegans*, which leads to excessive levels of branched-chain amino acids (BCAAs). We further show that BCAAs reduce a LET-363/mTOR-dependent neuro-endocrine signal, which we identify as DAF-7/TGFβ, and that impacts lifespan depending on its related receptors, DAF-1 and DAF-4, as well as ultimately on DAF-16/FoxO and HSF-1 in a cell-non-autonomous manner. The transcription factor HLH-15 controls and epistatically synergizes with BCAT-1 to modulate physiological ageing. Lastly and consistent with previous findings in rodents, nutritional supplementation of BCAAs extends nematodal lifespan. Taken together, BCAAs act as periphery-derived metabokines that induce a central neuro-endocrine response, culminating in extended healthspan.

## Abstract (web summary)

Organismal ageing is driven by conserved biological processes. Here the authors build on a comparative RNA-seq analysis in three model organisms to demonstrate that the gene, *bcat-1*, which catalyses the degradation of branched-chain amino acids, regulates lifespan in worms.

## Graphical abstract

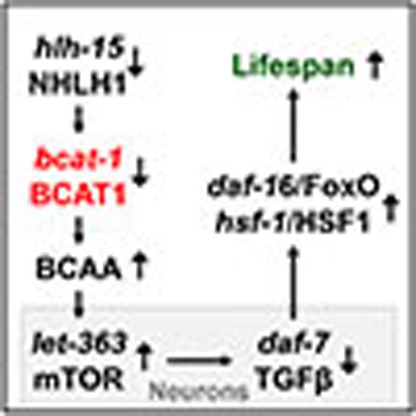

While the process of ageing has been fascinated humankind for several thousands of years, interventions that reproducibly delay physical decline have first been described less than a century ago^1^,^2^. With the rise of genetic methods, specific pathways that control the process of ageing have been identified and analysed, giving rise to novel approaches to improve quality of life at higher age^3^,^4^.

The first systematic studies to identify random mutations associated with longevity were performed in the model organisms *Caenorhabditis elegans*^2^. Subsequently, other organisms including *Saccharomyces cerevisiae*, *Drosophila melanogaster*, rodents and more recently several fish models were employed to dissect genetic pathways linked to physiological ageing. The best-studied one is the insulin/IGF-1 signalling pathway that has been identified in *C. elegans* to extend lifespan^5^,^6^. This role has then been extended to other organisms including *Drosophila*^7^,^8^ and rodents^9^,^10^,^11^. Given its evidently conserved nature, it was anticipated and subsequently confirmed that this pathway may impact human longevity as well^12^,^13^,^14^. While this and few other signalling pathways mediated by AMP-activated protein kinase (AMPK)^15^, mammalian target of rapamycin (mTOR)^16^,^17^, sirtuins^18^ or reactive oxygen species^19^,^20^ are evolutionary conserved, multiple others appear restricted to individual model organisms with little or no impact to mammals nor humans.

Concomitantly, unbiased screening approaches to identify ageing-associated genes and pathways have been performed for single model organisms indicating that ∼1 per cent of coding genes may impact the lifespan of nematodes^21^,^22^. Subsequently and to identify conserved pathways that may apply to several species, two invertebrate model organisms have been compared^23^, and genes identified in *S. cerevisiae* have been, at least in part, found to be conserved in *C. elegans*^24^.

We here have extended these approaches by comparing ageing-related gene expression patterns in three different organisms, namely *C. elegans*, zebrafish and mice, to identify ageing-related regulations of gene expression levels. We identify a crucial step for the catabolism of branched-chain amino acids (BCAAs), encoded by a gene named branched-chain amino acid transferase 1 (*bcat-1*) to be consistently regulated in three different organisms, and dissect the signalling role of BCAAs in *C. elegans* to promote increased healthspan.

## Results

### Identification of ageing-related genes in three species

**Figure 1:**
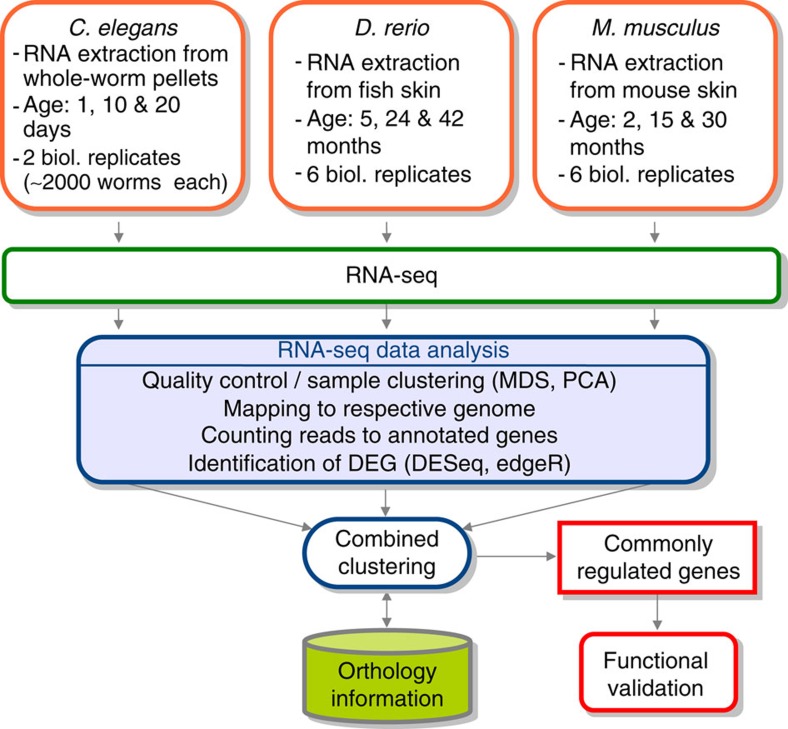
Sample acquisition and data processing scheme of the trans-species screening approach. RNAs of each sample were sequenced. After passing quality control and sample clustering, the sequences were mapped to the referring genome. The number of reads of the resulting annotated genes were used for statistical evaluation. Commonly regulated genes over the three species were subsequently tested individually for putative impact on lifespan in *C. elegans*.

**Figure 2:**
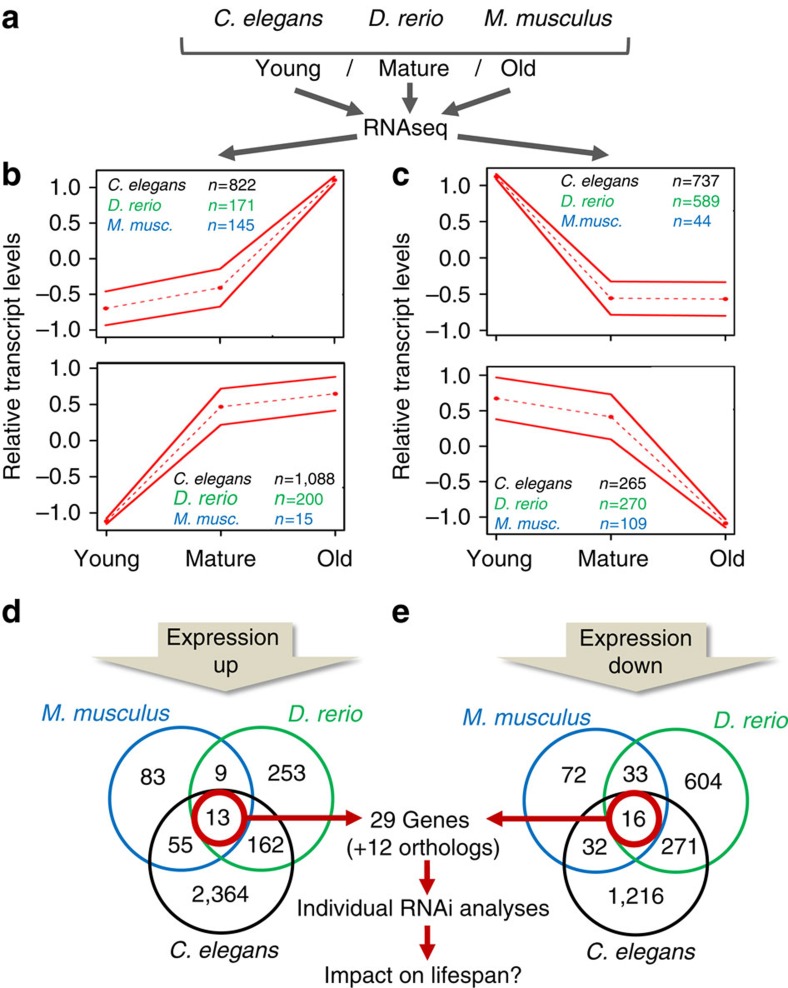
A trans-species screening approach to identify ageing-associated genes. (**a**) Depicts species subjected to RNA extraction at three different ages. (**b**) Depicts relative RNA transcript levels uniformly upregulated during physiological ageing. (**c**) Depicts relative RNA transcript levels uniformly downregulated during physiological ageing. (**d**,**e**) Show results of Venn analysis from genes identified in **b** and **c**, respectively. *C. elegans* results are shown in black, *D. rerio* in green and *M. musculus* in blue.

We have studied three well-established model organisms, the invertebrate nematode *C. elegans*, the vertebrate fish *Danio rerio* and the mammalian *Mus musculus* strain C57BL/6J, to identify genes that are similarly regulated on a transcriptional level during physiological ageing. We obtained skin samples from individual zebrafish and mice, as well as pellets containing ∼2,000 *C. elegans* Bristol N2, at three different ages (see Fig. 1 for details). RNA was extracted from these samples and subjected to Illumina next-generation sequencing (RNA-seq). About 13–82 million reads were obtained for each individual sample (Supplementary Data 1,2,3). Data analysis had to be restricted to genes for which orthologs could be identified in all three species (*C. elegans*: 4,850; *D. rerio*: 6,064; *M. musculus*: 5,904; Fig. 1). Those with transcript levels showing statistically significant differences by both DESeq and edgeR at least between two time points or by the baySeq test over the three time points were regarded as differentially expressed genes (DEG; *C. elegans*: 3,608; *D. rerio*: 1,721; *M. musculus*: 339; Supplementary Data 1,2,3). All DEGs were combined and the expression profiles optimally clustered into six courses, two of which each showed global up- or downregulation with ageing, respectively (Fig. 2 and Supplementary Fig. 1). We identified 13 genes to be upregulated during ageing in all three species, while 16 genes were found to be downregulated (Fig. 2).

### RNAi-based validation of ageing-related genes in *C. elegans*

**Figure 3:**
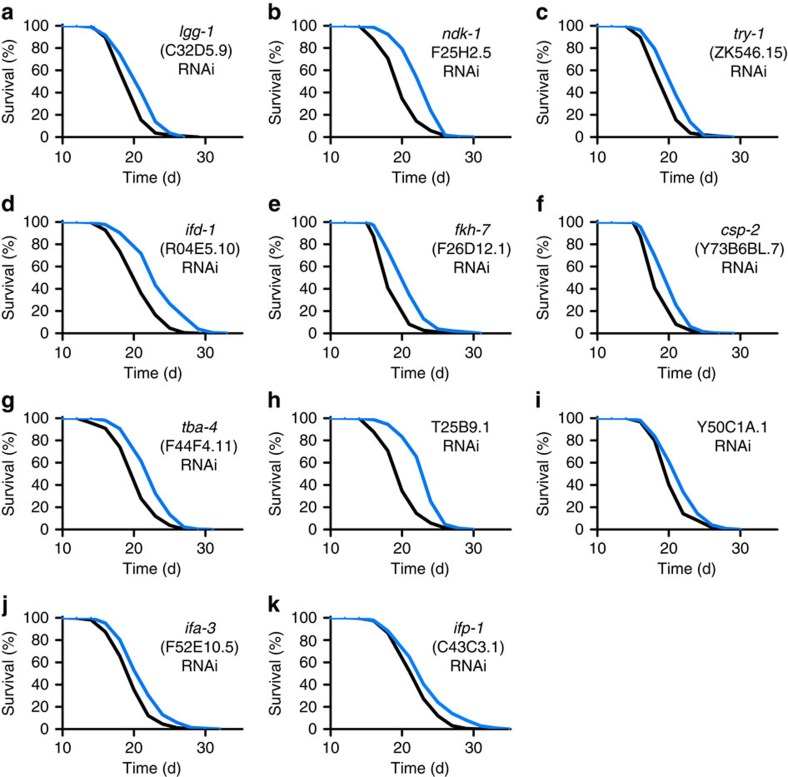
Lifespan analyses in *C. elegans* for validation of impact on ageing with significantly increased lifespan (≥5%). (**a**–**k**) depict lifespan assays following RNAi treatment during adult life with control vector (black) or RNAi against the respective gene (blue) starting at L4 larvae stage. For *P*-values and number of experiments see Supplementary Table 2. *Note that results for *bcat-1* have been omitted since shown subsequently*.

**Figure 4:**
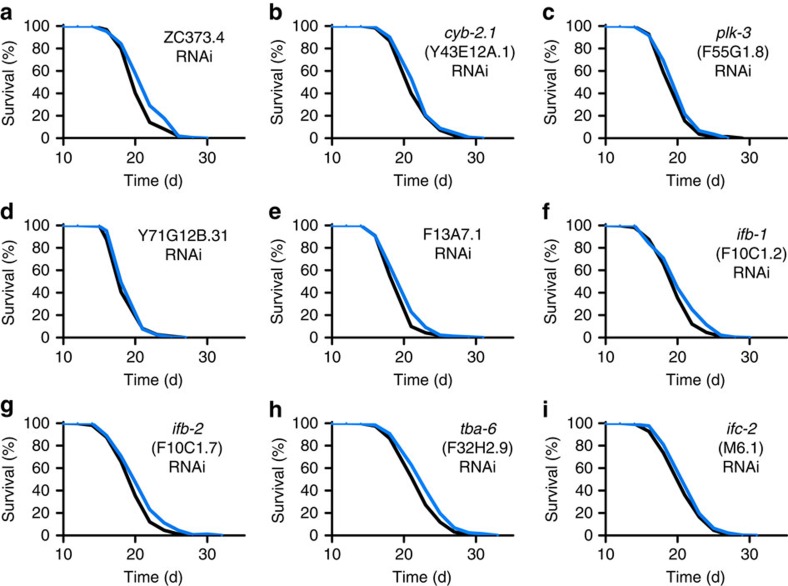
Lifespan analyses in *C. elegans* for validation of impact on ageing with significantly increased lifespan (<5%). (**a**–**i**) depict lifespan assays following RNAi treatment during adult life with control vector (black) or RNAi against the respective gene (blue) starting at L4 larvae stage. For *P*-values and number of experiments see Supplementary Table 2.

**Figure 5:**
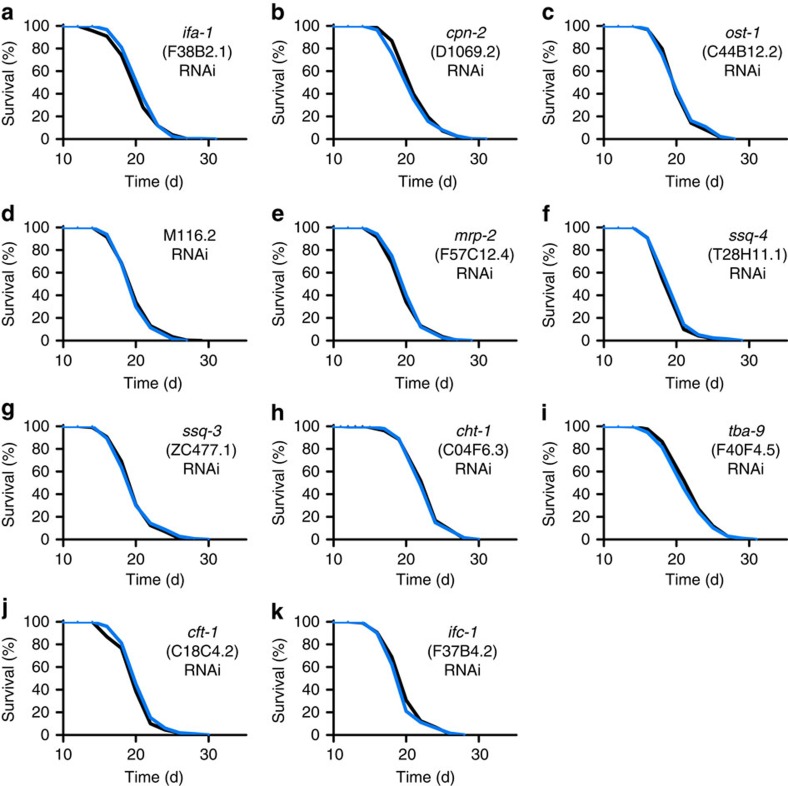
Lifespan analyses in *C. elegans* for validation of impact on ageing with no significant effect on lifespan. (**a**–**k**) depict lifespan assays following RNAi treatment during adult life with control vector (black) or RNAi against the respective gene (blue) starting at L4 larvae stage. For *P*-values and number of experiments see Supplementary Table 2.

**Figure 6:**
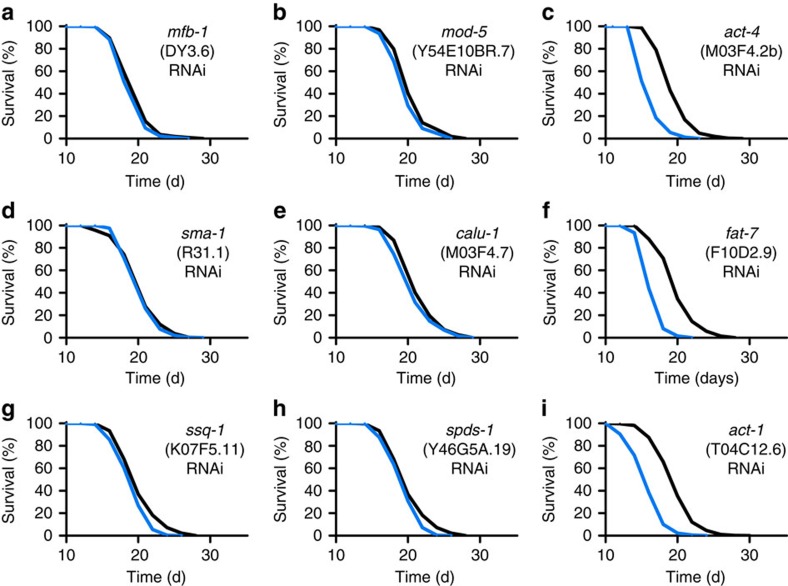
Lifespan analyses in *C. elegans* for validation of impact on ageing with significantly shortened lifespan. (**a**–**i**) depict lifespan assays following RNAi treatment during adult life with control vector (black) or RNAi against the respective gene (blue) starting at L4 larvae stage. For *P*-values and number of experiments see Supplementary Table 2.

All of these 29 genes and 12 additionally predicted paralogs (Supplementary Table 1) were individually targeted by feeding respective RNA interference (RNAi) to young adult worms, revealing that 30 out of 41 genes (73%) have an individual effect on life expectancy in worms (Table 1, Figs 3, 4, 5, 6 and Supplementary Table 2).

Out of these 41 genes, interference with 12 genes did extend mean lifespan by 5% or more (Table 1 and Supplementary Table 2). The most pronounced extension of lifespan was observed when applying RNAi against a crucial step for the catabolism of BCAAs, encoded by a gene named *bcat-1*.

### Impaired *bcat-1* expression extends *C. elegans* lifespan

**Figure 7:**
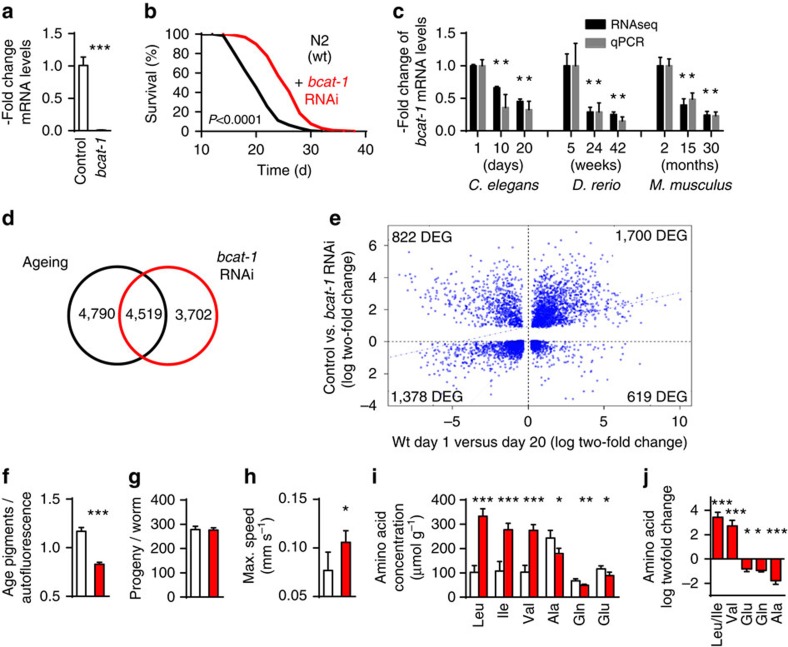
Validation and characterization of *bcat-1* as an ageing-related gene. (**a**) Shows *bcat-1* RNA levels in whole-worm RNA extracts after treatment of *C. elegans* with RNAi against *bcat-1* (red bar) versus control RNAi (open bar; *P*<0.001, Student's *t*-test, *n*=3). (**b**) Depicts the effect of *bcat-1* RNAi (red) versus control (black) on lifespan (*P*<0.0001, log-rank test, *n*=3). (**c**) Depicts qPCR results (grey) in comparison with RNA-seq results (black; samples as depicted in Fig. 1c; *P*<0.05 versus first time point; one-way ANOVA, *n*=3). (**d**) shows a Venn analysis of transcripts that are regulated by physiological ageing (blue) and *bcat-1* RNAi treatment (red), respectively. (**e**) Shows transcript levels as in **d** quantitatively (*P*<0.001, correlation, *n*=3). (**f**–**l**) Depict *bcat-1* RNAi-treated (red) versus control (open) nematodes regarding (**f**) ageing pigments (*P*<0.001, Student's *t*-test, *n*=8), (**g**) progeny (*P*=0.7, Student's *t*-test, *n*=10), (**h**) maximum crawling speed (*P*<0.05, Student's *t*-test, *n*=5), as well as changes in whole-worm amino acid concentrations as determined by (**i**) HPLC (*P<0.05, **P<0.01, ***P<0.001 versus control, one-way ANOVA, *n*=4), and (**j**) mass spectrometry (*P<0.05, **P<0.01, ***P<0.001 versus control, one-way ANOVA, *n*=4). Error bars represent the means±s.d.

Applying RNAi against *bcat-1* did abolish expression of the gene (Fig. 7a) and extended mean lifespan by 25% and maximum lifespan by 19%, reflecting the strongest effect of all genes identified (Fig. 7b, Figs 3, 4, 5, 6 and Supplementary Table 2). We next reanalysed our RNA samples using quantitative PCR (qPCR) to validate the RNA-seq results, and confirmed the downregulation of *bcat-1* transcript levels during physiological ageing in all three species (Fig. 7c). Additional support for the important role of BCAT-1 during ageing comes from an analysis of differentially regulated metabolic pathways during ageing in *C. elegans*, in which we found that the degradation of BCAAs, whose first step is catalysed by BCAT-1, was the most significantly downregulated pathway (26 out of 29 BCAA-metabolizing reaction steps, Supplementary Table 3). Moreover, published metabolomics-based evidence suggest that BCAAs are upregulated in long-lived *daf-2* nematodes in a *daf-16*-dependent manner ‘making them strong candidates for being causally involved in longevity'^25^ while a putative role of *bcat-1* has not been analysed in this regard. Taken together these findings suggest that *bcat-1* expression may promote ageing, and that ageing organisms may endogenously downregulate this gene, potentially to counteract ageing across species.

We next applied RNA-seq to nematodes exposed to *bcat-1* RNAi for 5 days, and analysed the number of genes consistently regulated during physiological ageing (Fig. 2) as well as in the state of *bcat-1* impairment. We found that physiological ageing and *bcat-1* impairment affects DEGs similarly where 45.8% (physiological ageing) and 54% (*bcat-1* RNAi) of the respective DEGs overlapped (Fig. 7d). Moreover, we found that physiological ageing and *bcat-1* RNAi up- or downregulate the same DEGs, respectively: while 1700 DEGs were up- and 1378 DEGs were downregulated in the same direction, only 619 and 822 DEGs were regulated in an opposing manner (Fig. 7e), respectively. Lastly, statistical analysis revealed a highly significant (MonteCarlo, *P*=0.00099) correlation between DEGs of normal ageing versus *bcat-1* RNAi, suggesting a global functional relevance of BCAT-1 for the process of physiological ageing.

### Impairing *bcat-1* expression promotes *C. elegans* healthspan

We next aimed at quantifying a number of ageing-associated parameters, including the accumulation of previously established ageing pigments^26^, which was observed to be reduced in 13-days-old nematodes exposed to *bcat-1* RNAi (Fig. 7f). While interventions to extend lifespan typically reduce fecundity, we did not observe such effect of *bcat-1* RNAI treatment (Fig. 7g and Supplementary Fig. 2a). In addition, we quantified maximum movement speed of nematodes and found impairment of *bcat-1* expression to increase this parameter (Fig. 7h). Hence, reduced *bcat-1* expression extends lifespan without affecting fecundity, reduces ageing pigments and promotes physical activity, consistent with increased health.

### Impairing *bcat-1* expression increases BCAAs

We next questioned whether blocking *bcat-1* expression would affect accumulation of its substrates, namely L-leucine, L-isoleucine and L-valine. Consistent with the biochemical role of the enzyme BCAT-1, all three BCAAs were found to be increased by 158% or more (L-val+167%, L-ile +158%, L-leu +225%), while L-alanine, L-glutamine and L-glutamate were found to be slightly decreased (Fig. 7i), possibly resembling anaplerotic refuelling of the Krebs cycle, while other amino acids remained unchanged (Supplementary Fig. 2b). The increase in BCAAs was independently confirmed using mass spectrometry-based metabolomics (Fig. 7j). The latter methodology in addition indicated a metabolic shift in *bcat-1*-impaired worms (Supplementary Data 4), further corroborated by RNA-seq pathway analysis of such nematodes (upregulated: neuro-active ligand-receptor interaction (cel04080); downregulated: TGFβ signalling pathway (cel04350), oxidative phosphorylation (cel00190), BCAA degradation (cel00280)), both in comparison with wild-type controls, at an age of 5 days.

### Increasing BCAA levels promotes *C. elegans* longevity

**Figure 8:**
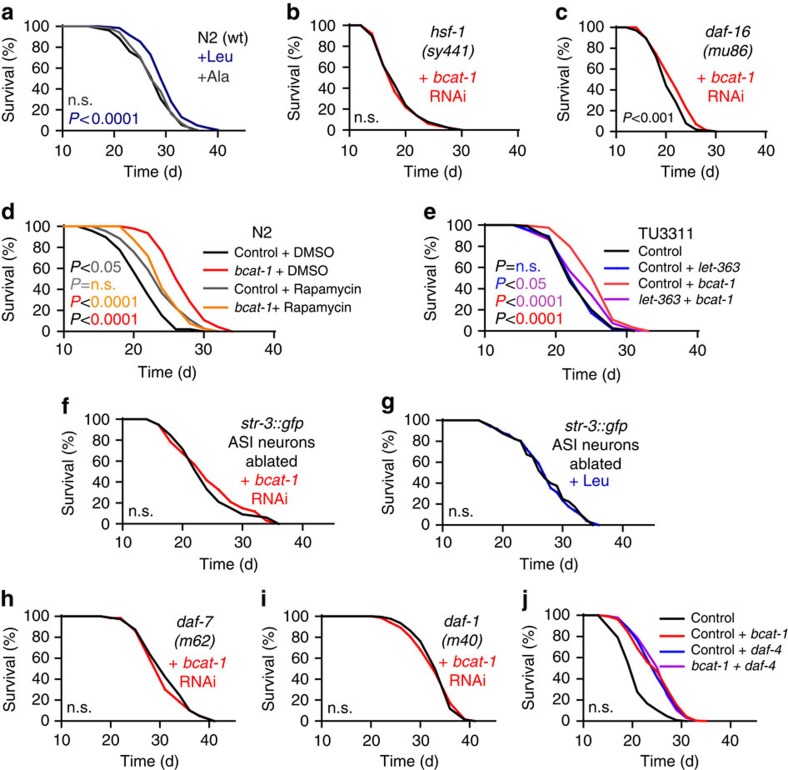
Increased BCAAs act through neuronal LET-363/mTOR and peripheral DAF-7/TGFβ signaling to extend lifespan. (**a**) Shows lifespan data of wild-type nematodes without treatment (black), as well as, exposure to L-alanine (grey, 5 mM) and L-leucine (blue, 5 mM) for their entire adult lifespan, maintained on non-metabolizing bacteria (*P*=0.24 for L-ala and *P*<0.0001 for L-leu both versus control, log-rank test, *n*=3). (**b**,**c**) Show effects of *bcat-1* RNAi (red) on lifespan in strains mutant for (**b**) *hsf-1* (*P*=0.67, log-rank test, *n*=3) and (**c**) *daf-16* (*P*<0.001, log-rank test, *n*=3). (**d**) Depicts the effects of the mTOR-inhibitor rapamycin (100 μM) on wild-type worms (grey versus black) versus the lack of effect of non-neuronal *bcat-1* RNAi in the presence of rapamycin (100 μM), all on lifespan (*P*<0.05 for control RNAi/rapamycin versus control RNAi/DMSO, *P*=0.07 for *bcat-1* RNAi/rapamycin versus control RNAi/rapamycin, *P*<0.0001 for *bcat-1* RNAi/rapamycin versus *bcat-1* RNAi/DMSO, *P*<0.0001 for *bcat-1* RNAi/DMSO versus control RNAi/DMSO, log-rank test, *n*=3). (**e**) Depicts the effects of neuronal *bcat-1* RNAi on lifespan in the presence (purple) and absence (red) of neuronal RNAi against *let-363*/mTOR (*P*=45 for control/*let-363* RNAi versus control RNAi, *P*<0.05 for control/*let-363* RNAi versus *let-363*/*bcat-1* RNAi, *P*<0.0001 for control/*bcat-1* RNAi versus *let-363*/*bcat-1* RNAi, *P*<0.0001 for control RNAi versus control/*bcat-1* RNAi, log-rank test, *n*=3). (**f**,**g**) Depict the lack of effect of (**f**) peripheral *bcat-1* RNAi (*P*=0.73, log-rank test, *n*=3) and (**g**) L-leucine supplementation (*P*=0.96, log-rank test, *n*=3) on an ASI-ablated reporter strain. (**h**–**j**) Show the lack of effect of *bcat-1* RNAi on strains mutant for (**h**) *daf-7* (*P*=0.15, log-rank test, *n*=3) and (**i**) *daf-1* (*P*=0.84, log-rank test, *n*=3), as well (**j**) in the co-presence (purple) or absence (red) of RNAi against *daf-4* (*P*=0.8 for control/*bcat-1* RNAi versus *daf-4*/*bcat-1* RNAi, *P*<0.0001 for all treatments versus control, log-rank test, *n*=3). For *P*-values and number of repetitions see Supplementary Table 4.

Based on the findings on increased BCAA levels (Fig. 7i,j), we then exposed wild-type N2 nematodes to the BCAA L-leucine at a concentration of 5 mM, while L-alanine served as a control. While L-alanine had no effect on *C. elegans* lifespan, L-leucine did promote longevity (Fig. 8a, Supplementary Table 4, applies to all subsequent lifespan analyses) however to a lesser extent than *bcat-1* RNAi (Fig. 7b). Similar results were obtained for the remaining BCAAs, namely L-isoleucine (Supplementary Fig. 2c) and L-valine (Supplementary Fig. 2d). These findings suggest that impaired *bcat-1* expression may promote lifespan, at least in part, by increasing organismal levels of different BCAAs.

We next tested whether and to which extent known transcriptional executers of lifespan-extending interventions may contribute to the phenotype by performing epistasis experiments. While the NRF2/SKN-1 pathway appears dispensable for the *bcat-1* RNAi- as well as the L-leucine-mediated lifespan extension (Supplementary Fig. 2e,f), both FoxO/Dauer formation 16 (DAF-16) and heat-shock factor 1 (HSF-1) appear to be epistatically relevant, since impairment of *hsf-1* abolished lifespan extension (Fig. 8b, Supplementary Fig. 2g ), and impaired *daf-16* expression reduced it almost completely (Fig. 8c and Supplementary Fig. 2h). Moreover, application of *bcat-1* RNAi to nematodes increases phosphorylation of the *C. elegans* equivalent of the murine Ser326 residue of HSF-1 (Supplementary Fig. 2i–l).

### *Bcat-1*-mediated longevity depends on mTOR activation

Quantifying individual amino acid concentrations following *bcat-1* impairment (Fig. 7i) also indicated a global increase (+38.4%) in organismal free amino acids. Increased levels of amino acids activate the mTOR pathway, or its *C. elegans* ortholog *let-363*, respectively. Inhibition of this pathway is known to extend lifespan in different species^17^, being in apparent conflict with our current findings (Figs 7b and 8a, Supplementary Fig. 2c,d). We therefore repeated the experiments depicted in Fig. 7b in the presence of the mTOR-inhibitor rapamycin and the respective solvent control. As previously observed^27^, rapamycin extended lifespan of *C. elegans* (Fig. 8d). Interestingly, the effect of *bcat-1* on lifespan was reduced by chemically inhibiting LET-363 to the same extent as lifespan was increased by rapamycin alone (Fig. 8d). This unexpectedly indicates that *bcat-1* disruption acts lifespan-extending in a *let-363*-dependent manner (Fig. 8d) paralleled by increased BCAAs and global amino acid levels (Fig. 7i,j).

### Neuronal mTOR signalling transduces *bcat-1* impairment

Treatment with RNAi is known to affect all tissues of the wild-type Bristol N2 strain except for neurons, while small molecule-based compounds like rapamycin also act on this latter cell type. This together with the findings summarized above raised the hypothesis that non-neuronal *bcat-1* interference would generate a cell-non-autonomous small-molecule signal that exerts its lifespan-extending effects in the neuronal compartment in a *let-363*-dependent manner. To test this, we used the *C. elegans* TU3311 strain known to respond to RNAi feeding by preferentially impairing expression of the corresponding gene in neurons only^28^. Impairing expression of *let-363* in neurons only had no effect on *C. elegans* lifespan (Fig. 8e), while the effect of reducing *bcat-1* in neurons had a strongly reduced, albeit still detectable effect (Fig. 8e). Interestingly, impairing expression of both *bcat-1* and *let-363* in neurons in parallel almost completely abolished the effect of *bcat-1* RNAi on lifespan (Fig. 8e). Given the fact that mTOR transduces availability of anabolic substrates and specifically the BCAA L-leucine in the murine hypothalamus^16^,^29^, we next analysed a possible involvement of the nematodal ASI neurons which are considered the hypothalamus equivalent in *C. elegans*. To test this, we laser-ablated these two neurons in nematodes and found the effect of *bcat-1* RNAi (Fig. 8f) and L-leucine feeding (Fig. 8g) on lifespan to be abolished, while sham-treated worms still responded to the respective intervention (Supplementary Figs 3a, b). Altogether this indicates that peripheral BCAAs activate neuronal *let-363* as well as ASI neuron-specific pathways to exert a lifespan-extending response.

### Hypothalamic TGFβ signalling executes *bcat-1* impairment

Given this potentially ASI-specific response (Fig. 8d,e) as well as the RNA-seq-based pathway analysis (Fig. 7l), we next studied the nematodal paralog of mammalian TGFβ, *daf-7*, as a putative candidate to explain the BCAA-dependent activation of *let-363* in neurons, also since *daf-7* expression is restricted to ASI neurons^30^ and to the *daf-16* pathway^31^,^32^, while hypothalamic TGFβ is linked to ageing in mammals^33^. When exposing a strain with a constitutive inactivation of *daf-7*, namely *daf-7 (m62)*, to *bcat-1* RNAi no effect of this treatment was observed (Fig. 8h) opposing the effect in wild-type nematodes (Fig. 7b). This indicates that ASI-specific release of DAF-7 may execute the effect of *bcat-1* impairment on lifespan. To further support this, we epistatically tested whether the absence of known DAF-7-receptor genes, namely *daf-1* and *daf-4*, would affect *bcat-1* RNAi-mediated lifespan extension. Indeed, the impairment of either *daf-1* (Fig. 8i) or *daf-4* (Fig. 8j) expression completely inhibited the *bcat-1* RNAi effects on lifespan: while *daf-1*-mutated worms were long-lived, and so were nematodes treated with *daf-4* RNAi, addition of *bcat-1* RNAi would not extend lifespan further, indicating that *bcat-1* and *daf-1/daf-4* epistatically share a downstream pathway. Notably and since *daf-4* RNAi does not affect neurons, while *daf-7* expression is limited to ASI neurons^30^, this also indicates that this TGFβ/*daf-7* signal qualifies as a cell-non-autonomous feedback loop linking peripheral signals that activate neuronal (and potentially ASI-specific) mTOR back to the periphery.

### Overexpression of *bcat-1* impairs lifespan and fecundity

**Figure 9 f9:**
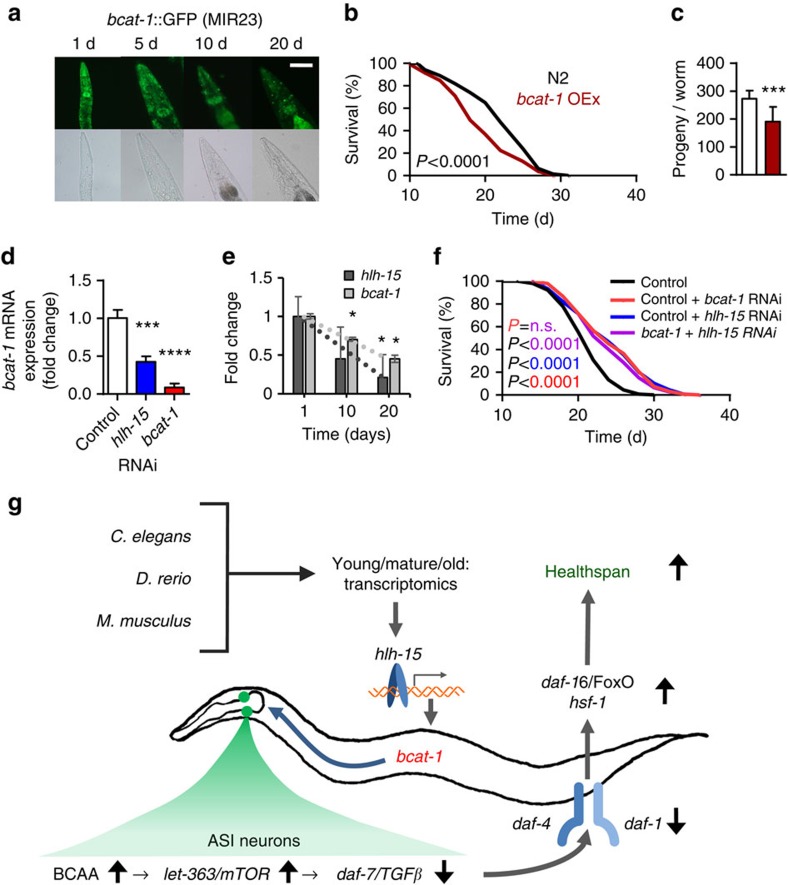
Transcriptional control of *bcat-1*-mediated regulation of lifespan. (**a**) Fluorescent microscopy of nematodes transgenically expressing *bcat-1* fused to GFP under the control of the endogenous *bcat-1* promoter, at different ages (scale bar, 100 μm). (**b**) Shows the effect of *bcat-1* overexpression on lifespan (*P*<0.001, log-rank test, *n*=3). (**c**) Depicts fertility as reflected by the number of eggs (***P<0.001, Students's *t*-test, *n*=10). (**d**) Depicts transcript levels of *bcat-1* in the presence of control RNAi (white), RNAi against *hlh-15* (blue) and *bcat-1* (red) in wild-type nematodes (***P<0.001, ****P<0.0001 versus control, one-way ANOVA, *n*=4). (**e**) Shows transcript levels of *bcat-1* (black) and *hlh-15* (grey) during physiological ageing in wild-type worms (*P<0.05, one-way ANOVA, Pearson correlation Fisher *Z*, *P*=0.053, *n*=3). (**f**) Depicts the effects of control RNAi (black), control RNAi combined with *bcat-1* RNAi (red; *P*<0.0001 versus control, log-rank test, *n*=3), control RNAi combined with *hlh-15* RNAi (blue; *P*<0.0001 versus control, log-rank test, *n*=3) and *bcat-1* RNAi combined with *hlh-15* RNAi (purple, epistasis; *P*<0.0001 versus control, *P*=0.08 versus control/*bcat-1* RNAi, log-rank test, *n*=3) on *C. elegans* lifespan. (**g**) Summarizes the effects of *hlh-15*-controlled *bcat-1* expression, or BCAA supplementation, on neuronal *let-363*/*daf-7* signaling looping back to the periphery to control lifespan. For *P*-values and number of repetitions see Supplementary Table 4. Error bars represent the means±s.d.

We next questioned whether overexpression of *bcat-1* would have opposing effects on lifespan, particularly in comparison to states of impaired expression, as shown above (Fig. 7b). To this purpose, we expressed a *bcat-1*-complementary DNA (cDNA) 3′-/C-terminally fused to a green fluorescent protein (GFP)-cDNA under the control of the endogenous *bcat-1* promoter. Fluorescent microphotographs indicated a reduction of BCAT-1 expression with increasing age (Fig. 9a), consistent with the gene expression levels of the endogenous *bcat-1* in wild-type worms (Fig. 7c). Importantly and opposing the findings on *bcat*-1 RNAi (Fig. 7b), overexpression of *bcat-1* shortens *C. elegans* lifespan (Fig. 9b), further supporting a regulatory role in the regulation of lifespan. Moreover, overexpression of *bcat-1* decreased fertility of nematodes (Fig. 9c) that, from an evolutionary perspective, suggests a selective advantage of low *bcat-1* expression levels, notably independent of ageing.

### HLH-15 is a transcriptional regulator of *bcat-1*

Based on the fact that reduced versus increased levels of BCAT-1 exhibit opposing effects on lifespan (Figs 7b versus 9a, [Fig f9]), we next questioned whether and how *bcat-1* expression may be systemically controlled during physiological ageing. We *in silico* analysed a 1,000 base-pair promoter fragment upstream of the *bcat-1* start codon, and identified a transcription factor named helix-loop-helix factor 15 (*hlh-15)* to (i) show the highest (*n*=3) number of binding sites within this promoter fragment and to have (ii) the highest *P*-value for binding probability (Markov Chain model, *P*=8.7e-07; Supplementary Table 5).

To confirm the *in silico* prediction that HLH-15 may control the expression of *bcat-1*, we applied RNAi against *hlh-15* to wild-type nematodes and found expression of *bcat-1* to be reduced by >50% (Fig. 9d). When analysing the RNA-seq data obtained from *C. elegans* during physiological ageing (Fig. 2), we found expression levels of *bcat-1* (Fig. 9e, Pearson *r*=−0.9964) and *hlh-15* (Fig. 9e, Pearson *r*=−0.9693) to be correlated by trend (Pearson *P*=0.0537), suggesting that HLH-15 may regulate *bcat-1* expression during physiological ageing. To lastly test this, we have performed the corresponding epistasis experiments: RNAi against *bcat-1* or against *hlh-15*, respectively, extended nematodal lifespan to the exact same extent (Fig. 9f). Co-application of both RNAis did show the same effect as each RNAi individually (Fig. 9f), altogether suggesting that HLH-15 and BCAT-1 may actively synergize in the regulation of physiological ageing (Fig. 9g).

## Discussion

Increased BCAA catabolism and specifically increased activity of the corresponding enzyme, BCAT, has been linked to various pathological states, including accelerated growth of malignant gliomas^34^, decreased sepsis survival^35^ and increased accumulation of liver fat^36^, the latter being linked to a number of metabolic diseases^37^. Consistently, systemic disruption of one *BCAT* isoform^38^, namely *BCATm*, in mice increases energy expenditure and reduces body weight^39^.

More specifically, L-leucine is capable of reducing food uptake in rodents when injected into the hypothalamus, notably depending on neuronal mTOR (refs 16, 29). By contrast, others have linked hypothalamic BCAAs to increased insulin resistance, providing a potential link to type 2 diabetes^40^,^41^. Since nematodal ASI neurons are considered the homologue of the mammalian hypothalamus^42^, and ablation of these two neurons abolishes the effect of both L-leucine (Fig. 8g) and *bcat-1* RNAi (Fig. 8f), the effect of increasing organismal L-leucine levels on lifespan (Fig. 7b,i,j) may be translatable into mammals. Indeed, feeding increased amounts of BCAAs to middle-aged mice did improve healthspan depending on induction of endothelial nitric oxide synthase^43^, an enzyme not known in *C. elegans*^44^. Moreover, other hypothalamic signals have been recently linked to the regulation of lifespan in rodents^45^,^46^, while mTOR signalling has not been tested in this regard.

Global inhibition of mTOR is generally considered to promote lifespan in different species^17^ including *C. elegans*^27^ (also confirmed by our control findings, Fig. 8d). By contrast and unexpectedly, we here find that activation of mTOR in nematodal neurons due to a peripheral BCAA signal promotes lifespan (Fig. 7e) and parameters of healthspan (Fig. 7f,h) without affecting fecundity (Fig. 7g). Exerting the BCAA signal in neurons only still exerts an effect on lifespan, however to a reduced extent (Fig. 8e). Activation of mTOR is known to induce HSF-1 (ref. 47), and the latter suppresses *daf-7* (ref. 48), consistent with our findings which link mTOR activation to suppression of *daf-7*/TGFβ signalling (Figs 7l and 8h–j). Since *daf-7* is expressed in ASI neurons only^30^, while its receptors *daf-1* and *daf-4* act in the periphery^49^, *daf-7*/TGFβ may now be considered to act cell-non-autonomous as a neuro-endocrine hormone that impairs lifespan in otherwise healthy animals in response to evolutionary conserved pathways and amino acid signals derived thereof (Fig. 9g).

## Methods

### Chemicals

All chemicals were obtained from Sigma-Aldrich (Munich, Germany) unless stated otherwise.

### *C. elegans* strains and maintenance

*C. elegans* strains used for this publication were provided by the *Caenorhabditis Genetics Center* (University of Minnesota, USA). Nematodes were grown and maintained on Nematode growth media (NGM) agar plates at 20 °C using *E. coli* OP50 bacteria as food source^50^. After plates were poured and dried, they were sealed and stored at 4 °C. Freshly prepared *E. coli* were spotted on plates on the previous evening and allowed to dry and settle overnight. For all experiments we used Bristol N2 wild-type except the following we received from CGC: EU31 *skn-1(zu135*), PS3551 *hsf-1(sy441*), CF 1038 *daf-16(mu86)*, CX3596 *kyIs128[str-3::GFP]; lin-15B(n765)*, TU3311 *uIs60[unc-119p::YFP+unc-119p::sid-1]*, SJ4100 *zcIs13[hsp-6::GFP]*, DR62 *daf-7(m62)*and DR40 *daf-1(m40).*

### Sample preparation for physiological worm ageing experiments

Freshly prepared bacteria OP50 were spotted on 10 cm NGM agar plates on the previous evening and allowed to dry and settle overnight. Synchronized, young adult worms (64 h after synchronization) were transferred to fresh plates using S-Buffer^51^. For maintaining synchronized populations in long-term experiments, worms were daily washed off the plates to 15 ml tubes, allowed to settle and washed until the supernatant was free of progeny. The clean worm pellet was transferred to freshly prepared treatment plates. Worms were pelleted and frozen at an adult age of 1, 10 and 20 days.

### RNAi-mediated gene knockdown and compound treatment

For RNAi gene knockdown experiments we applied E. coli HT115 to the worms as previously described^52^. The clones for *act-1,daf-4, cyb-2.1, ssq-1, F13A7.1, ssq-4, T25B9.1, ifa-1, ifa-3, ifb-2, ifc-2, ifd-1, ndk-1, plk-3, try-1, calu-1, fat-7, spds-1, cpn-2, tba-4, tba-6, mfb-1, lgg-1 and hlh-15* RNAi were obtained from feeding RNAi ORF library v1.1 (Thermo Fisher Scientififc, Waltham, MA, USA). The clone for *bcat-1, let-363, cht-1, ost-1, ifb-1, ifp-1, sma-1, tba-9, mod-5*, Y50C1A.1 and ZC373.4 derived from Ahringer library (Source BioScience, Nottingham, UK). The remaining clones were generated from PCR products of genomic DNA and were cloned into control vector L4440 using its EcoRV restriction site. Primer sequences are listed in Supplementary Table 6. All (that is, also commercially obtained) clones were sequenced prior use.

The bacteria were spotted on NGM plates containing additionally 1 mM isopropyl-β-D-thiogalactoside, 100 μg ml^−1^ ampicillin and, if required, 12.5 μg ml^−1^ tetracycline (all from Applichem, Darmstadt, Germany). After plates were poured and dried, they were sealed and stored at 4 °C. Freshly prepared bacteria were spotted on plates on the previous evening and allowed to dry and settle overnight. Incubations with compounds started 64 h after synchronization of the population, by washing the synchronized, young adult worms and then transferring them to the respective treatment plates using S-Buffer^51^. For maintaining synchronized populations in long-term experiments, worms were daily washed off the plates to 15 ml tubes, allowed to settle and washed until the supernatant was free of progeny. The clean worm pellet was transferred to freshly prepared treatment plates.

For compound treatments, all agar plates were prepared from the same batch of NGM agar, whereas treatment plates were supplemented with L-leucine, L-alanine (both 5 mM final concentration) or water as solvent control. Rapamycin was used in a concentration of 100 μM and dimethylsulphoxide (DMSO) served as solvent control. For all experiments with amino acid supplementation only heat-inactivated bacteria were used to avoid influences on bacterial metabolism. The bacteria were heat-inactivated for 45 min at 65 °C in a shaking incubator and resuspended and concentrated (20 × ) in S-buffer containing 5 μg ml^−1^ and 10 mM MgSO_4_. The experimental procedures are the same as described above for RNAi treatment experiments.

### Nematodal lifespan assays

All lifespan assays were performed at 20 °C according to standard protocols and as previously described^52^. Briefly, a *C. elegans* population was synchronized as described above at day 0 of the lifespan. 64 h after egg preparation around 100 nematodes were manually transferred to fresh incubation plates containing the respective compounds. Experiments were conducted in triplicates. For the first 10 days, worms were transferred every day and afterwards every second day. Nematodes that show no reaction to gently stimulation were scored as death. Those animals that crawled off the plates or display non-natural death due to internal hatching were censored.

### *D. rerio*

Zebrafish of the TüAB strain were kept in groups of 20–30 animals under standard husbandry conditions. Skin tissue from male zebrafish at the age of 5 months (*n*=12), 24 months (*n*=12) and 42 months (*n*=6) was dissected and stored in RNAlater (Qiagen, Hilden, Germany) at −80 °C. Total RNA was isolated with TRIzol (Life Technologies, Darmstadt, Germany) according to the instructions of the manufacturer.

### *M. musculus*

Young (2 months), mature (15 months) and aged (30 months) old mice were deeply anaesthetized with isoflurane anaesthesia (2.5% in a mixture of 3:1 N_2_O:O_2_). One square cm hairless abdominal skin was taken and snap frozen. The isolated skin was homogenized in 500 μl QIAzol (Qiagen) by subsequently adding 100 μl chloroform. Following phase separation, the aqueous phase was transferred into a fresh tube, then 0.16 volume NaAc (2 M, pH 4.0) and 1.1 volume isopropanol were added. The RNA was precipitated by centrifugation and the pellet was washed with 75% ethanol. Total RNA was resuspended in 20 μl water and stored at –80 °C until use.

### Breeding and housing conditions

The study was carried out on male C57BL/6J mice (Jackson Laboratories). Animals of given ages were raised in our own facilities. All animal procedures were approved by the local government (Thueringer Landesamt, Bad Langensalza, Germany) and conformed to international guidelines on the ethical use of animals. All mice were maintained in a specific pathogen-free environment at room temperature (22 °C) at 68% humidity and light/dark (12 h/12 h) cycles with access to water and food (V1534-300, SsniffSpezialdiäten GmbH, Soest, Germany), *ad libitum* and were tested negative for parasites and other routine pathogens.

### RNA-seq

RNA integrity was determined by Agilent's Bioanalyzer 2100 (with RNA 7500 kit, both Agilent Technologies, Santa Clara, CA, USA).

*M. musculus and C. elegans physiological ageing samples*. Total RNA (2.5 μg) was used with Illumina'sTruSeq RNA sample prep kit v2 following the manufacturer's instruction. Illumina 50 bp single reads (SR) were obtained using the HiSeq2000 by multiplexing four samples per lane. Sequencing resulted in around 40–50 mio reads per sample. The sequence information was extracted using Illumina's supported CASAVA v1.7 as FastQ format.

*D. rerio*. Total RNA (5 μg) was used for preparation of multiplex libraries using Illumina's mRNA-Seq sample prep kit (Illumina, San Diego, CA, USA) following the manufacturer's instruction. Libraries were sequenced in one per lane using Illumina's Genome Analyzer (GAIIx) in SR mode creating reads with a length of 76 bp. Sequencing resulted in around 30 mio reads per samples. Sequence information was extracted in FastQ format using the Illumina's supported GA-Pipeline v1.5.

*C. elegans bcat-1 RNAi perturbation samples*. Total RNA (1 μg) was used for library preparation using TruSeq RNA sample prep kit v2 following the manufacturer's instruction. Sequencing was done on a HiSeq2500 in SR/50 bp/high output mode. Libraries were multiplexed in five per lane. Sequencing resulted in around 35-45 mio reads per sample. The sequence information was extracted using Illumina's supported bcl2FastQ v1.8.4 as FastQ format.

### RNA-seq data analysis

*Normal ageing samples*. The resulting FastQ files were mapped using Bowtie^53^ versus the respective genomic sequences and a splice site data set created using UCSC's RefSeq annotation for each species. Counting of uniquely mapped reads and assignment to RefSeq transcripts/genes was performed using R Statistical Language and Bioconductor. Afterwards, reads per kilobase transcript per million reads (RPKM) values^54^ were calculated for each transcript and gene from the corresponding RefSeq annotation. For each species DEG were identified using the DESeq^55^, edgeR^56^, and the baySeq^57^ packages. Those genes showing statistically significant differences (FDR adjusted *P*<0.05) by DESeq and edgeR at least between two time points or by the baySeq test over the three time points were regarded as DEGs (*C. elegans*: *n*=3,608; *D. rerio*: *n*=1,721; and *M. musculus*: *n*=339). Next, orthology relations between genes of the three species were obtained using EnsemblCompara and the orthology R package^58^ to facilitate the cross-species comparison. Since the individual species exhibit different lifespan, the gene expression time courses were rescaled, followed by a combined fuzzy c-means clustering with the exponent *m*=2 of the orthologous DEG expression profiles^59^, to identify common time courses. The optimal number of six clusters was estimated by the vote of several cluster validity indices, which capture different aspects of a clustering structure^60^. By intersecting the resulting clustered gene sets with the orthology relations extracted previously, gene sets with similar temporal pattern across all three species were identified.

For the analysis of differentially regulated metabolic pathways in the normal ageing samples, *C. elegans* genes were mapped to metabolic pathways using the PathwayTools software^61^. A reaction was assumed differentially regulated if at least one enzyme catalysing this reaction was significantly differentially expressed between young and old worms. Pathways were assumed significantly differentially regulated if a hypergeometric test of differentially regulated reactions yielded a FDR adjusted *P*-value<0.05.

*C. elegans bcat-1 RNAi perturbation samples*. FastQ files were mapped using Tophat (v2.0.6)^62^ versus the reference genome WBcel235.74 obtained from Ensembl. Uniquely mapped reads were counted for all genes using featureCounts^63^. RPKM values were computed using exon lengths provided by featureCounts and the sum of all mapped reads per sample. DEG were identified using the DESeq and edgeR. Generally applicable gene set enrichment for pathway analysis (GAGE)^64^ was used to detect significantly regulated Kyoto Encyclopedia of Genes and Genomes (KEGG) pathways (FDR adjusted *P*<0.05).

To evaluate whether the probability for the intersection of two gene expression sets is significantly different from the intersection of random sets we applied a Monte Carlo-based test. We first randomly generated subsets out of our two universe lists of all genes which were measured in both data sets according the number of genes of our real data sets. Then, we counted the number of genes in these random intersections for 1,000 iterations which resulted in a distribution of random intersection values. If the observed intersection value was >95% quantile we considered our intersection of genes to be significant.

### Reverse transcriptase qPCR (RT-qPCR)

Reverse transcription of RNA into cDNA was generated with the iScript cDNA synthesis kit (Bio-Rad, Munich, Germany) and qPCR was carried out with the SybrGreenERqPCRSupermix (Life Technologies) for iCycler. All PCR reactions were performed in triplicates and negative controls were always included. Ct-values of *bcat-1* were normalized to two reference genes (for *C. elegans*: *cdc-42* and *pmp-3*; for zebrafish: *tbp* and *insra*; for mouse: *gapdh* and *hmbs*; see Supplementary Table 7 for sequences). Determination of age-specific expression and statistical analysis was carried out using the relative expression software tool^65^.

*Bcat-1 RNAi perturbation C. elegans*. Adult wild-type worms were treated for 48 h with control vector L4440 or *bcat-1* RNAi, respectively.

### Laser ablation of ASI neurons

The laser ablation of ASI neurons was performed as described^42^,^66^ and as follows: for focused laser ablation the output laser beam of a UV pulsed laser (diode-pumped, Q-Switched Frequency-Tripled Laser System: Triton; TEM00, 349 nm; maximum power 1 W, pulsed; repetition rate: 1–1,000 Hz; pulse width: <15 ns; pulse energy: adjustable from 1 to 200 μJ; Spectra Physics, Darmstadt, Germany) was expanded by a telescope system and was coupled into a confocal laser scanning microscope (LSM510) via epi-fluorescence illumination path. The laser beam was focused into the object plane by a Zeiss Plan-Neofluar 100/1.30 oil objective (spot diameter <500 nm) after reflection by a dielectric mirror (Laser Optik, Germany). The dielectric mirror is placed on the empty laser scanning position of the fluorescence reflector slider and transmits the scanning lasers as well as the emitted fluorescence and reflects the pulsed laser beam of Triton. Thus the imaging functions of the laser scanning microscope (LSM) are not reduced. Before entering the microscope, laser pulse energy was reduced by a gradient position dependent attenuator (Laser Optik) to 80%.

Paralyzed post-L4 nematodes that expresses GFP from a ASI-specific *str-3* promoter *(kyIs128[str-3::gfp*]) were irradiated by several laser pulses at laser energy of 4 μJ. The live-cell damage was recorded and controlled by time series function of the Zeiss laser scanning microscope which is equipped with an Argon ion laser and emission filter sets for the detection of EGFP signals (BP530/20) and the Zeiss LSM software version 3.2. Immediately after ablation, worms were transferred to the corresponding treatment plates and analysed for life expectancy according to the described lifespan protocol above.

### Protein quantification

Protein content in nematodes and cells was determined by the Bradford method^67^ or the bicinchoninic acid (BCA) method^68^. Assays were performed in 96-well plates using commercial available kits (Bio-Rad Laboratories AG, Cressier, Switzerland, and Thermo Scientific, Waltham, MA, USA). Absorbance was measured in a micro-plate reader (Fluostar Optima, BMG Labtech, Offenburg, Germany).

### Determination of amino acid concentrations by HPLC

Frozen worm pellets have been ground with 200 μl PBS (pH=7.4) and de-proteinized with sulfo-salicylic acid (final concentration 2%; Sigma-Aldrich, St Louis, MO, USA). The cell debris and protein precipitate were separated by spinning. For the measurements 30 μl of supernatant was used.

Determination of amino acid concentration was carried out on a Biochrome 30+ Amino Acid Analyzer (Biochrom, Cambridge, UK) following manufacturer's instructions. This standard method in clinical diagnostics is based on separation on a retention column with lithium citrate buffer of different pH. The post-column derivatization with ninhydrin enables photometric detection at 570 and 440 nm. Samples were normalized to protein content as described above.

### Non-targeted metabolomics analysis

Metabolite extracts were prepared from worms by smashing (Schütt rotation homogenizer) and heating to 70 °C for 10 min in ethanol. After centrifugation the supernatant was stored at −80 °C until use.

Non-targeted analysis of the metabolome was performed by flow injection analysis-time of flight mass spectrometry on an Agilent 6550 QTOF instrument^69^. All samples were injected in duplicates. Ions were annotated based on their accurate mass and the KEGG has reference list allowing a tolerance of 0.001 Da. Unknown ions and those annotated as adducts were discarded.

### Locomotion analysis

Worms were synchronized and treated for 10 days with control vector or *bcat-1* RNAi as described above. After 10 days four to five worms were picked from RNAi plates and released on fresh plates without bacteria. We defined the refuge behaviour induced velocity as maximum speed. Directly after release 1 min movie clips were recorded with a Leica system (Leica M165FC with Leica camera DFC 3000 G). Subsequently, the videos were analysed using parallel worm tracker software developed by Goodman Lab which tracks based on centroid position of the worms^70^. The pixel to distance ratio was calibrated. For each treatment five independent videos were used.

### Age pigment analysis

Age pigments in *C. elegans* reflect biological age^26^. Worms were synchronized and treated for 10 days with control vector or *bcat-1* RNAi starting at L4 larvae stage. On day 10 worms were washed off the plates and distributed on 8 wells of a 96-well plate (Bioswisstec 96-well CG black with glass bottom, art. no.: 5241). The fluorescence of the age pigments was measured using a fluorescence plate reader (FLUOstar Omega, BMG Labtech, Offenburg, Germany; exciatition: 340-10 nm, emission: 440-80 nm; gain: 1,844). We normalized the age pigment fluorescence to the stable auto-fluorescence signal of the worms (filters: excitation: 290-10 nm, emission: 330-10 nm; gain: 1800) as described^26^.

### Fertility assay

For determination of fertility we synchronized nematodes as previously described^52^. Single L4 larvae were transferred on single plates carrying RNAi bacteria and subsequently every 24 h to fresh plates. Progeny were allowed to hatch and counted. For every condition 10 worms were used.

### *Bcat-1* overexpression

We combined a 1 kb endogenous promoter with a *bcat-1*-cDNA C-terminally fused to GFP, as well as an *unc-119* rescue gene, and implemented the construct into nematodes using ballistic transformation techniques as described before^71^. The generated transgenic lines were selected for stable integration. The resulting strain named MIR23 carrying the integrated construct *risIs[Pbcat-1::bcat-1::gfp+unc119]* has been used for experiments.

### Immunoblotting

Nematodes were washed three times with ice-cold S-buffer and pellets were shock-frozen in liquid nitrogen. Frozen pellets were grinded in a nitrogen-chilled mortar and suspended in phosphate buffer containing protease and phosphatase inhibitors (Complete protease inhibitor cocktail (Roche, Penzberg, Germany) and additionally 2 mM sodium fluoride, 2 mM sodium orthovanadate, 1 mM phenylmethylsulphonyl fluoride and 2 mM EDTA). Extracts were sonicated three times and centrifuged for 7 min at 12,000*g* at 4 °C. Supernatants were used for protein quantification, and an aliquot was boiled in Laemmli buffer and applied to SDS–polyacrylamide gel electrophoresis. Antibodies against phospho-HSF-1 (1:1,000, pSer326, Enzo, order number ADI-SPA-902-D) and alpha-tubulin (1:2,000, clone DM1A; Sigma-Aldrich; order number T6199) were used.

### Promoter analyses

The search for transcription factor binding sites was done within the proximal *bcat-1* promoter region 1 kb upstream of the predicted start codon. Therefore, a FASTA file containing the promoter region of *bcat-1* was created using WormMart^44^. Next, the remaining sequence file was scanned for one or more matches to the position-specific scoring matrices of all available transcription factors using the matrix scan function of the pattern-matching programme RSAT (regulatory sequence analysis tools)^72^. The position-specific scoring matrices contains the nucleotide frequency at each position within the binding sites and were obtained from the databases Transfac and Jaspar^73^,^74^,^75^. The threshold *P*-value, which indicates the risk of false–positive predictions, was set to 0.0001.

### Statistical analyses

Data are expressed as means±s.d. unless otherwise indicated. Statistical analyses for all *C. elegans* data except lifespan assays were performed by Student's *t*-test (unpaired, two-tailed) or one-way analysis of variance (ANOVA) after testing for equal distribution of the data and equal variances within the data set. For comparing significant distributions between different groups in the lifespan assays and stress resistance assays, statistical calculations were performed using JMP software version 10 (SAS Institute Inc., Cary, NC, USA) applying the log-rank test. The comparison of correlation between *bcat-1* and *hlh-15* expression over age was performed by applying Fisher z transformation of the Pearson correlation coefficients.

## Additional information

**Accession codes:** RNA-Seq data were deposited in gene expression omnibus (GEO) under accession codes GSE46051 (normal ageing of *C. elegans*), GSE46916 (normal ageing of *D. rerio and M. musculus*) and GSE60672 (*bcat-1* RNAi perturbation in *C. elegans*).

**How to cite this article:** Mansfeld, J. *et al*. Branched-chain amino acid catabolism is a conserved regulator of physiological ageing. *Nat. Commun.* 6:10043 doi: 10.1038/ncomms10043 (2015).

## Supplementary Material

Supplementary Information Supplementary Figures 1-3 and Supplementary Tables 1-7

Supplementary Data 1 Differentially expressed genes *C. elegans*

Supplementary Data 2 Differentially expressed genes *D. rerio*

Supplementary Data 3 Differentially expressed genes *M. musculus*

Supplementary Data 4 Differentially expressed genes: control RNAi versus *bcat-1* RNAi

## Figures and Tables

**Table 1:** Effects of individual RNAis on *C. elegans* lifespan.

**Effect on lifespan**	**Upregulated genes**	**Downregulated genes**
Shortened	*sma-1, mod-5, mfb-1*	*calu-1, spds-1, ssq-1, act-4, fat-7, act-1*
Unchanged	*mrp-2*, *cft-1*, M116.2, *cht-1*	*cpn-2, tba-9, ifa-1, ost-1, ssq-4, ssq-3, ifc-1*
Extended <5%	Y71G12B.31, ZC373.4	*plk-3, tba-6, ifb-2*, F13A7.1, *ifb-1, ifc-2, cyb-2.1*
Extended ≥5%	*fkh-7, csp-2, lgg-1*, Y50C1A.1	*bcat-1*, T25B9.1, *ndk-1, ifd-1, tba-4, ifa-3*, *try-1, ifp-1*
