# Supplementary material for: Branched-chain amino acid catabolism is a conserved regulator of physiological ageing
Source: Nat Commun. 2015 Dec 1;6:10043. doi: 10.1038/ncomms10043 (PMC4686672; doi:10.1038/ncomms10043)
Supplement: Supplementary Information — Supplementary Figures 1-3 and Supplementary Tables 1-7 [file ncomms10043-s1.pdf]

Supplementary Figure 1

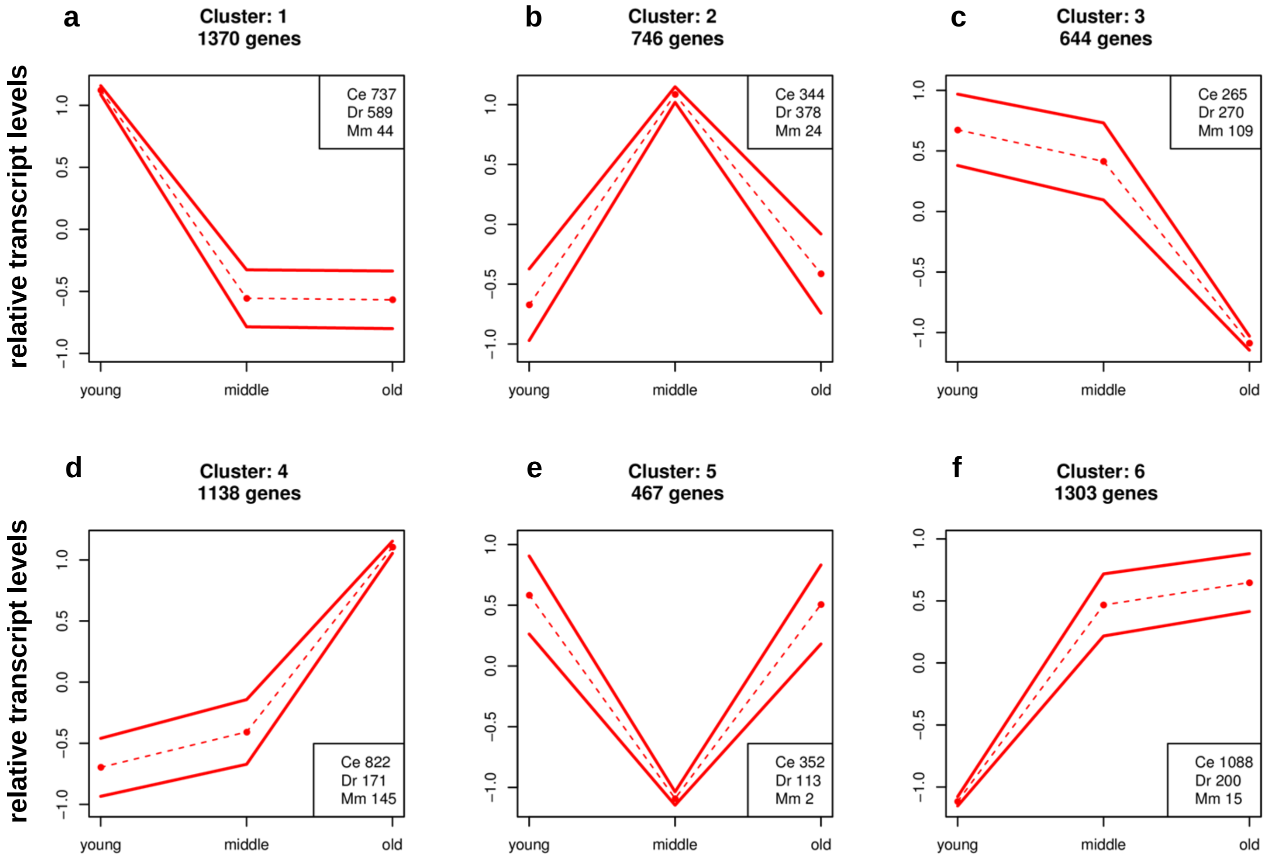

Supplementary Figure 1: Cluster analysis of similarly regulated genes in all three species analyzed. The pattern of the 6 analyzed clusters (**panels a-f**)of gene regulation during aging. Number of genes regulated in each species is specified in the referring box (Ce – *Caenorhabditis elegans*, Dr – *Danio rerio*, Mm – *Mus musculus*). **(a and c)** depict genes uniformly upregulated during aging, **(d and f)** genes uniformly downregulated. **(b and e)** depict genes that were intermittentely upregulated **(b)** or downregulated **(e)**.

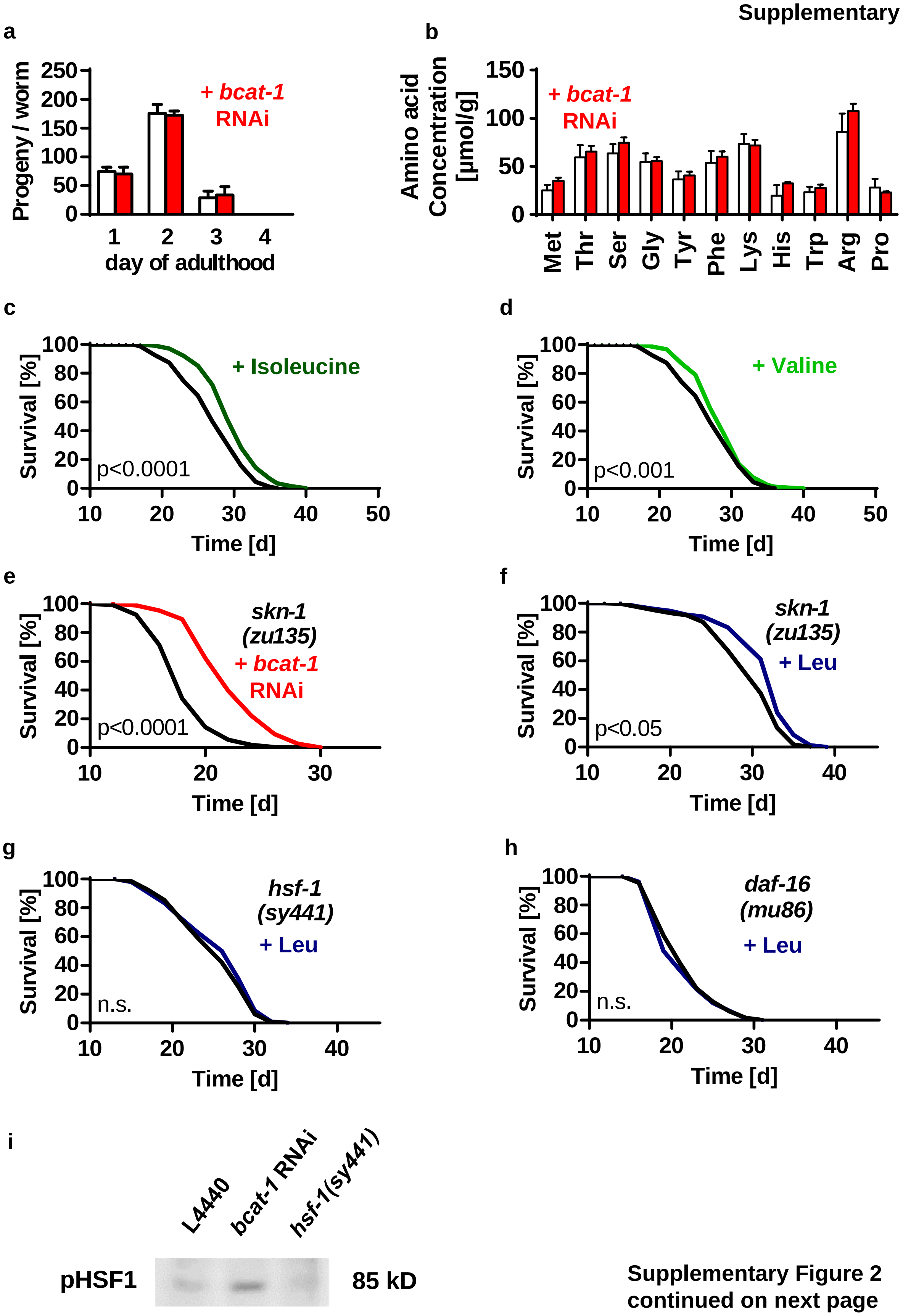

Supplementary Figure 2
continued on next page

j

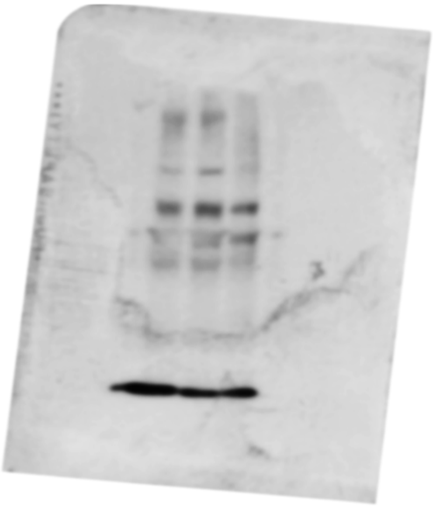

k

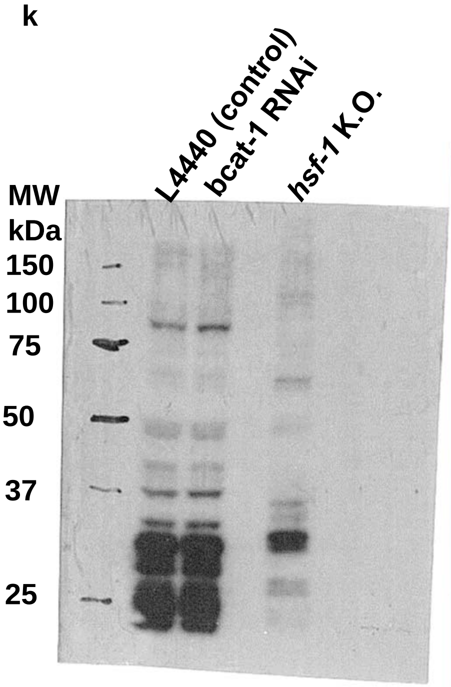

l

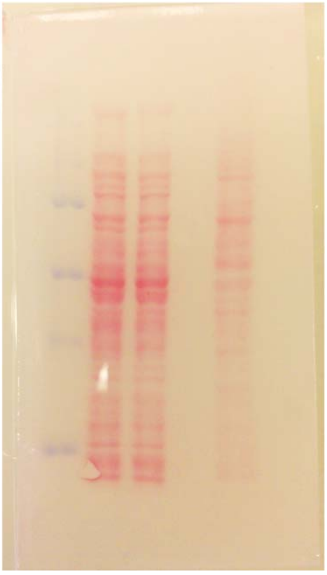

Supplementary Figure 2: Increased BCAAs act through neuronal *let-363*/mTOR and peripheral *daf-7*/TGFβ signaling to extend lifespan (additional panels). (a) shows effect of *bcat-1* RNAi on number of hatched eggs laid within in the first four days of adulthood by singled worms compared to control (open bar) (all p>0.05, one-way ANOVA, n=10). (b) Lack of changes on depicted free amino acid concentrations in whole-worm as determined by HPLC following treatment of *bcat-1* RNAi (red) versus control (open bars) (all p>0.05, one-way ANOVA, n=4). (c) and (d) depict lifespan effect of 5 mM L-isoleucine (dark green) (p<0.0001, log-rank test, n=3) and L-valine (bright green) (p<0.001, log-rank test, n=3) vs. control (black) in N2 wildtype worms. **(e)** and **(f)** depict lifespan effect of *bcat-1* RNAi (red) (p<0.0001, log-rank test, n=3) and 5 mM L-leucine (blue) (p<0.05, log-rank test, n=3) vs. control (black) in *skn-1(zu135)* K.O. mutants. (g) and (h) depict the lack of lifespan extending effects of 5 mM L-leucine (blue) (p=0.059 in *skn-1* K.O., p=0.26 in *daf-16* K.O., log-rank test, n=3) vs. control (black) in *hsf-1(sy441)* and *daf-16(mu86)* K.O. mutants. (i) shows increased phosphorylation status of HSF-1 in *bcat-1* RNAi treated N2 wildtype worms, (j) the corresponding complete film, (k) an independent experiment as in (i), and (l) the corresponding membrane.

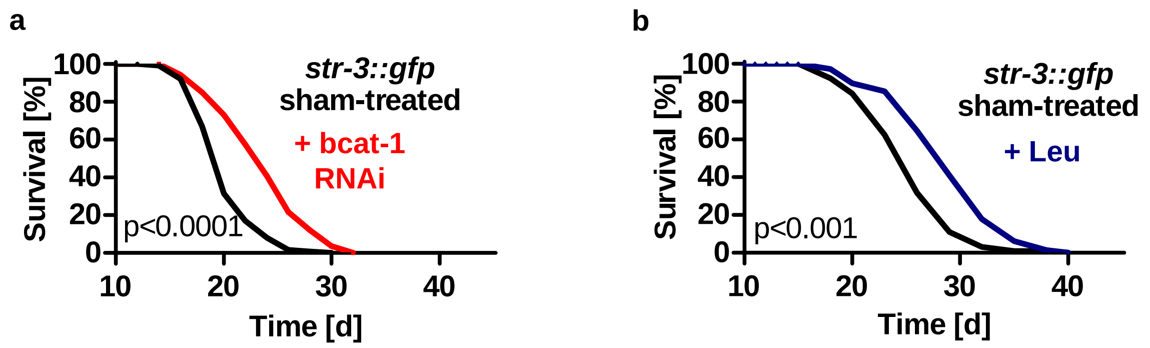

Supplementary Figure 3: Increased BCAAs act through neuronal *let-363*/mTOR and peripheral *daf-7*/TGFβ signaling to extend lifespan (additional panels - continued).
(a) and **(b)** show effect on lifespan of *bcat-1* RNAi (red) ($p<0.0001$, log-rank test, n=3) and 5 mM L-leucine (blue) ($p<0.001$, log-rank test, n=3) vs. control (black) in sham-treated *str-3::gfp* (GFP-labeled ASI neurons).

Supplementary Table 1: List of genes consistently regulated during physiological aging

Mouse gene name	*C.elegans* orthologs Gene name	*C.elegans* orthologs Sequence name
Acta1/Actc1	*act-1*	T04C12.6
	act-4	M03F4.2b
Bcat1	*bcat-1*	K02A4.1
Ccnb1	*cyb-2.1*	Y43E12A.1
Eln	F13A7.1	F13A7.1
	ssq-1	K07F5.11
	ssq-3	ZC477.1
	ssq-4	T28H11.1
Gcat	T25B9.1	T25B9.1
Lmnb1	*ifa-1*	F38B2.1
	ifa-3	F52E10.5
	ifb-1	F10C1.2
	ifb-2	F10C1.7
	ifc-1	F37B4.2
	ifc-2	M6.1
	ifd-1	R04E5.10
	ifp-1	C43C3.1
Nme1	*ndk-1*	F25H2.5
Plk1	*plk-3*	F55G1.8
Prss53	*try-1*	ZK546.15
Rcn3	*calu-1*	M03F4.7
Scd3	*fat-7*	F10D2.9
Sparc	*ost-1*	C44B12.2
Srm	*spds-1*	Y46G5A.19
Tagln3	*cpn-2*	D1069.2
Tuba8	*tba-4*	F44F4.11
	tba-6	F32H2.9
	tba-9	F40F4.5
Abcc2	*mrp-2*	F57C12.4
	cft-1	C18C4.2
Casp1/Casp4	*csp-2*	Y73B6BL.7
Clmn	*sma-1*	R31.1
Fbxo32	*mfb-1*	DY3.6
Foxp2	*fkh-7*	F26D12.1
Gabarapl1	*lgg-1*	C32D5.9
Gyg	M116.2	M116.2
Mylk2	ZC373.4	ZC373.4
Ovgp1	*cht-1*	C04F6.3
Ptpn22	Y71G12B.31	Y71G12B.31
Slc6a4	*mod-5*	Y54E10BR.7
Zranb1	Y50C1A.1	Y50C1A.1
n=29	n=41	

SupplementaryTable 2: Lifespan assay details of validation experiments of 41 aging-related genes

Gene Name	Gene Name	Life Span	p-value	Mean Life Span	Mean Life Span	Maximum Lifespan	Maximum Life Span	Number
(M.musculus)	*(C.elegans*	(+/0/-)	(versus control)	days ±SEM	(%)	75th percentile (days) ±SEM	75th percentile (%)	Nematodes
	control			20.68		23.6 ±0.9		250
Bcat1	*bcat-1*	+	< 0.0001	25.85	125.00	28.0 ±0.0	118.64	250
	control			20.48 ± 0.2		22.4 ± 0.4		285
Nme1	*ndk-1*	+	< 0.0001	23.10 ± 0.2	112.78	25.2 ± 0.5	112.50	388
	control			20.48 ± 0.2		22.4 ± 0.4		285
Scd3	*fat-7*	-	< 0.0001	16.96 ± 0.1	82.81	18.0 ± 0.0	80.36	316
	control			20.48 ± 0.2		22.4 ± 0.4		285
Gcat	T25B9.1	+	< 0.0001	23.45 ± 0.1	114.49	25.2 ± 0.5	112.50	394
	control			20.06 ± 0.1		21.0 ± 0.0		302
Fbxo32	*mfb-1*	-	< 0.01	19.55 ± 0.3	97.48	21.0 ± 0.0	100.00	305
	control			20.06 ± 0.1		21.0 ± 0.0		302
Gabarapl1	*lgg-1*	+	< 0.0001	21.22 ± 0.2	105.77	23.0 ± 0.0	109.52	384
	control			21.92 ± 0.1		23.0 ± 0.0		411
Rcn3	*calu-1*	-	< 0.01	21.28 ± 0.2	97.08	22.2 ± 0.5	96.52	311
	control			21.92 ± 0.1		23.0 ± 0.0		411
Tagln3	*cpn-2*	o	0.1559	21.53 ± 0.3	91.20	23.0 ± 0.0	100.00	291
	control			21.92 ± 0.1		23.0 ± 0.0		411
Ccnb1	*cyb-2.1*	+	< 0.01	22.46 ± 0.4	102.41	23.8 ± 0.5	103.48	399
	control			21.31 ± 0.3		22.2 ± 0.5		243
Tuba8	*tba-4*	+	< 0.0001	22.88 ± 0.2	107.37	25.0 ± 0.0	112.61	398
	control			21.31 ± 0.3		22.2 ± 0.5		243
Lmnb1	*ifa-1*	o	0.66	21.42 ± 0.2	100.49	22.6 ± 0.4	101.80	284
	control			21.31 ± 0.3		22.2 ± 0.5		243
Clmn	*sma-1*	-	< 0.05	20.33 ± 0.4	95.40	21.8 ± 0.5	98.20	340
	control			20.06 ± 0.1		21.0 ± 0.0		302
Prss53	*try-1*	+	< 0.0001	21.36 ± 0.2	106.50	23.0 ± 0.0	109.52	346
	control			20.54 ± 0.1		22.0 ± 0.0		307
Srm	*spds-1*	-	< 0.001	19.85 ± 0.2	96.63	20.8 ± 0.5	94.55	240
	control			20.54 ± 0.1		22.0 ± 0.0		307
Eln	*ssq-1*	-	< 0.0001	19.63 ± 0.2	95.56	21.2 ± 0.5	96.36	245
	control			20.06 ± 0.1		21.0 ± 0.0		302
Plk1	*plk-3*	+	< 0.05	20.57 ± 0.2	102.56	22.0 ± 0.5	104.76	218
	control			20.85 ± 0.2		22.0 ± 0.0		321
Sparc	*ost-1*	o	0.72	20.88 ± 0.1	100.16	22.0 ± 0.0	100.00	315
	control			20.85 ± 0.2		22.0 ± 0.0		321
Mylk2	ZC373.4	+	< 0.0001	21.74 ± 0.2	104.28	24.0 ± 0.6	109.09	318
	control			20.85 ± 0.2		22.0 ± 0.0		321
Slc6a4	*mod-5*	-	< 0.001	20.13 ± 0.1	96.54	21.6 ± 0.4	98.18	270
	control			20.85 ± 0.2		22.0 ± 0.0		321
Zranb1	Y50C1A.1	+	< 0.001	21.94 ± 0.2	105.22	24.0 ± 0.6	109.09	361
	control			20.33 ± 0.2		21.6 ± 0.4		377
Gyg	M116.2	o	0.32	20.21 ± 0.2	99.42	21.6 ± 0.4	100.00	384
	control			20.33 ± 0.2		21.6 ± 0.4		377
Abcc2	*mrp-2*	o	0.18	20.62 ± 0.2	101.47	22.0 ± 0.0	101.85	375

Supplementary Table 2 continued

Gene Name	Gene Name	Life Span	p-value	Mean Life Span	Mean Life Span	Maximum Lifespan	Maximum Life Span	Number
(M.musculus)	*(C.elegans)*	(+/0/-)	(versus control)	(days ±SEM)	(%)	(75th percentile (days) ±SEM)	(75th percentile (%)	Nematodes
	control			19.22 ± 0.1		21.0 ± 0.0		344
Casp1	*csp-2*	+	< 0.0001	20.67 ± 0.1	107.59	21.8 ± 0.5	103.81	401
	control			19.22 ± 0.1		21.0 ± 0.0		344
Foxp2	*fkh-7*	+	< 0.0001	21.27 ± 0.2	110.68	23.0 ± 0.0	109.52	394
	control			19.22 ± 0.1		21.0 ± 0.0		344
Ptpn22	Y71G12B.31	+	< 0.05	19.60 ± 0.1	101.98	21.0 ± 0.0	100.00	368
	control			19.88 ± 0.2		21.0 ± 0.0		276
Acta1	*act-4*	-	< 0.0001	16.54 ± 0.2	83.14	17.4 ± 0.4	82.86	342
	control			23.30± 0.12		24.0 ± 0.0		545
Ovgp1	*cht-1*	o	0.48	23.26± 0.1	99.83	24.0 ± 0.0	100.00	592
	control			19.79 ± 0.1		21.0 ± 0.0		370
Eln	*ssq-4*	o	0.06	20.11 ± 0.1	101.60	21.0 ± 0.0	100.00	372
	control			19.79 ± 0.1		21.0 ± 0.0		370
Eln	F13A7.1	+	< 0.001	20.45 ± 0.2	103.35	21.4 ± 0.4	101.90	374
	control			20.13 ± 0.2		21.6 ± 0.4		378
Lmnb1	*ifa-3*	+	< 0.0001	21.60 ± 0.3	107.30	23.6 ± 0.4	109.26	373
	control			20.13 ± 0.2		21.6 ± 0.4		378
Lmnb1	*ifb-2*	+	< 0.0001	20.95 ± 0.1	104.08	22.8 ± 0.5	105.56	374
	control			20.13 ± 0.2		21.6 ± 0.4		378
Lmnb1	*ifb-1*	+	< 0.01	20.77 ± 0.2	103.16	22.8 ± 0.5	105.56	292
	control			20.13 ± 0.2		21.6 ± 0.4		378
Acta1	*act-1*	-	< 0.0001	16.27 ± 0.1	80.80	18.0 ± 0.0	83.33	381
	control			22.43 ± 0.1		25.0 ± 0.0		350
Tuba8	*tba-6*	+	< 0.0001	23.38 ± 0.3	104.23	25.8 ± 0.5	103.20	365
	control			22.43 ± 0.1		25.0 ± 0.0		350
Tuba8	*tba-9*	o	0.16	22.02 ± 0.2	98.19	23.8 ± 0.5	95.20	365
	control			22.43 ± 0.1		25.0 ± 0.0		350
Lmnb1	*ifp-1*	+	< 0.0001	23.73 ± 0.4	105.81	26.2 ± 0.5	104.80	368
	control			21.25 ± 0.2		23.0 ± 0.0		400
Lmnb1	*ifc-2*	+	< 0.001	21.84 ± 0.1	102.76	23.0 ± 0.0	100.00	301
	control			21.25 ± 0.2		23.0 ± 0.0		400
Lmnb1	*ifd-1*	+	< 0.0001	23.87 ± 0.1	112.35	26.2 ± 0.5	113.91	389
	control			20.24 ± 0.1		22.0 ± 0.0		324
Lmnb1	*ifc-1*	o	0.12	19.92 ± 0.2	98.43	20.4 ± 0.4	92.73	296
	control			20.24 ± 0.1		22.0 ± 0.0		324
Eln	*ssq-3*	o	0.67	20.32 ± 0.1	100.40	21.6 ± 0.4	98.18	295
	control			20.68 ± 0.1		22.0 ± 0.0		295
Abcc2	*cft-1*	o	0.0686	21.00 ± 0.1	101.56	22.0 ± 0.0	100.00	302

Supplementary Table 3: Metabolic pathways differentially expressed during aging in *C. elegans*.

Gene ID	Reaction ID	Reaction name	Differentially expressed	
			Genes	Reaction
alh-8	1_2_1_27_RXN	Methylmalonate-semialdehyde dehydrogenase (acylating)	yes	yes
acdh-7	2_MEBUCOA_FAD_RXN	2-methylacyl-CoA dehydrogenase	no	yes
acdh-10	2_MEBUCOA_FAD_RXN	2-methylacyl-CoA dehydrogenase	no	yes
acdh-8	2_MEBUCOA_FAD_RXN	2-methylacyl-CoA dehydrogenase	yes	yes
acdh-3	2_MEBUCOA_FAD_RXN	2-methylacyl-CoA dehydrogenase	no	yes
K09H11.1	2_MEBUCOA_FAD_RXN	2-methylacyl-CoA dehydrogenase	yes	yes
B0250.5	3_HYDROXYISOBUTYRATE_DEHYDROGENASE_RXN	3-hydroxyisobutyrate dehydrogenase	yes	yes
F09F7.4	3_HYDROXYISOBUTYRYL_COA_HYDROLASE_RXN	3-hydroxyisobutyryl-CoA hydrolase	yes	yes
kat-1	ACACT1	Acetyl-CoA C-acetyltransferase	yes	yes
T02G5.7	ACACT1	Acetyl-CoA C-acetyltransferase	yes	yes
T02G5.4	ACACT1	Acetyl-CoA C-acetyltransferase	yes	yes
F35A2.7	ACACT10	acetyl-CoA:propanoyl-CoA 2-C-acetyltransferase	no	yes
B0303.3	ACACT10	acetyl-CoA:propanoyl-CoA 2-C-acetyltransferase	yes	yes
acdh-3	ACOAD9	isobutyryl-CoA dehydrogenase	no	no
T08B2.7	ECOAH9i	Tiglyl-CoA hydratase	no	yes
ech-7	ECOAH9i	Tiglyl-CoA hydratase	yes	yes
ech-6	ECOAH9i	Tiglyl-CoA hydratase	yes	yes
ech-5	ECOAH9i	Tiglyl-CoA hydratase	yes	yes
ech-3	ECOAH9i	Tiglyl-CoA hydratase	no	yes
ech-1	ECOAH9i	Tiglyl-CoA hydratase	no	yes
ard-1	HACD9	3-hydroxy-2-methylbutyryl-CoA dehydrogenase	yes	yes
F25B4.6	HMGCOAS	Hydroxymethylglutaryl-CoA synthase	no	no
Y71G12B.10	HMGL	Hydroxymethylglutaryl-CoA lyase	no	no
bcat-1	ILETA	Isoleucine transaminase	yes	yes
Y44A6D.5	ILETA	Isoleucine transaminase	no	yes
bcat-1	LEUTA	Leucine transaminase	yes	yes
Y44A6D.5	LEUTA	Leucine transaminase	no	yes
ech-7	METHYLACYLYLCOA_HYDROXY_RXN	Methylacrylyl-CoA hydratase	yes	yes
ech-6	METHYLACYLYLCOA_HYDROXY_RXN	Methylacrylyl-CoA hydratase	yes	yes
ech-5	METHYLACYLYLCOA_HYDROXY_RXN	Methylacrylyl-CoA hydratase	yes	yes
ech-3	METHYLACYLYLCOA_HYDROXY_RXN	Methylacrylyl-CoA hydratase	no	yes
ech-1	METHYLACYLYLCOA_HYDROXY_RXN	Methylacrylyl-CoA hydratase	no	yes
T08B2.7	METHYLACYLYLCOA_HYDROXY_RXN	Methylacrylyl-CoA hydratase	no	yes
ech-5	MGCH	Methylglutaconyl-CoA hydratase	yes	yes
ZK669.4	OIVD2	2-oxoisovalerate dehydrogenase (acylating)	yes	yes
tag-172	OIVD2	2-oxoisovalerate dehydrogenase (acylating)	no	yes
LLC1.3	OIVD2	2-oxoisovalerate dehydrogenase (acylating)	yes	yes
Y39E4A.3	OIVD2	2-oxoisovalerate dehydrogenase (acylating)	no	yes
ZK669.4	OIVD3	2-oxoisovalerate dehydrogenase	yes	yes
tag-172	OIVD3	2-oxoisovalerate dehydrogenase	no	yes
LLC1.3	OIVD3	2-oxoisovalerate dehydrogenase	yes	yes
Y39E4A.3	OIVD3	2-oxoisovalerate dehydrogenase	no	yes

Supplementary Table 3 continued

Gene ID	Reaction ID	Reaction name	Differentially expressed	
			Genes	Reactions
ZK669.4	RXN909_116	Branched-chain & alpha-keto acid dehydrogenase complex (for 4-mehtyl-2-oxopentanoate)	yes	yes
tag-172	RXN909_116	Branched-chain & alpha-keto acid dehydrogenase complex (for 4-mehtyl-2-oxopentanoate)	no	yes
LLC1.3	RXN909_116	Branched-chain & alpha-keto acid dehydrogenase complex (for 4-mehtyl-2-oxopentanoate)	yes	yes
Y39E4A.3	RXN909_116	Branched-chain & alpha-keto acid dehydrogenase complex (for 4-mehtyl-2-oxopentanoate)	no	yes
gta-1	RXN909_117	L-3-amino-isobutanoate:2-oxoglutarate aminotransferase	yes	yes
hacd-1	RXN909_15	(2S,3S)-3-hydroxy-2-methylbutanoyl-CoA:NAD+ oxidoreductase	yes	yes
F54C8.1	RXN909_15	(2S,3S)-3-hydroxy-2-methylbutanoyl-CoA:NAD+ oxidoreductase	no	yes
ech-9	RXN909_15	(2S,3S)-3-hydroxy-2-methylbutanoyl-CoA:NAD+ oxidoreductase	yes	yes
ech-8	RXN909_15	(2S,3S)-3-hydroxy-2-methylbutanoyl-CoA:NAD+ oxidoreductase	yes	yes
B0272.3	RXN909_15	(2S,3S)-3-hydroxy-2-methylbutanoyl-CoA:NAD+ oxidoreductase	yes	yes
ard-1	RXN909_15	(2S,3S)-3-hydroxy-2-methylbutanoyl-CoA:NAD+ oxidoreductase	yes	yes
acdh-7	RXN909_24	isobutyryl-CoA dehydrogenase	no	yes
acdh-10	RXN909_24	isobutyryl-CoA dehydrogenase	no	yes
acdh-8	RXN909_24	isobutyryl-CoA dehydrogenase	yes	yes
K09H11.1	RXN909_24	isobutyryl-CoA dehydrogenase	yes	yes
F54C8.1	RXN909_26	3-hydroxyisobutyrate dehydrogenase	no	yes
ech-9	RXN909_26	3-hydroxyisobutyrate dehydrogenase	yes	yes
B0272.3	RXN909_26	3-hydroxyisobutyrate dehydrogenase	yes	yes
hacd-1	RXN909_26	3-hydroxyisobutyrate dehydrogenase	yes	yes
ech-8	RXN909_26	3-hydroxyisobutyrate dehydrogenase	yes	yes
alh-9	RXN909_28	(S)-Methylmalonate semialdehyde:NAD+ oxidoreductase	yes	yes
R2RD2	RXN909_28	(S)-Methylmalonate semialdehyde:NAD+ oxidoreductase	no	yes
alh-1	RXN909_28	(S)-Methylmalonate semialdehyde:NAD+ oxidoreductase	yes	yes
K09H11.1	RXN909_34	isovaleryl-CoA dehydrogenase	yes	yes
acdh-8	RXN909_34	isovaleryl-CoA dehydrogenase	yes	yes
acdh-7	RXN909_34	isovaleryl-CoA dehydrogenase	no	yes
acdh-10	RXN909_34	isovaleryl-CoA dehydrogenase	no	yes
ivd-1	RXN909_35	3-Methylbutanoyl-CoA:(acceptor) 2,3-oxidoreductase	yes	yes
ech-7	RXN909_36	3-Hydroxyisopentyl-CoA hydro-lyase	yes	yes
T08B2.7	RXN909_36	3-Hydroxyisopentyl-CoA hydro-lyase	no	yes
ech-6	RXN909_36	3-Hydroxyisopentyl-CoA hydro-lyase	yes	yes
ech-5	RXN909_36	3-Hydroxyisopentyl-CoA hydro-lyase	yes	yes
ech-3	RXN909_36	3-Hydroxyisopentyl-CoA hydro-lyase	no	yes
ech-1	RXN909_36	3-Hydroxyisopentyl-CoA hydro-lyase	no	yes
F32B6.2	RXN909_37	3-Methylcrotonoyl-CoA:carbon-dioxide ligase (ADP-forming)	yes	yes
C05C10.3	RXN909_38	succinyl-CoA:acetoacetate CoA-transferase	yes	yes
bcat-1	VALTA	Valine transaminase	yes	yes
Y44A6D.5	VALTA	Valine transaminase	no	yes

Supplementary Table 4: Lifespan assay details of all remaining experiments

strain	genotype	RNAi treatment	treatment	p-val vs.	p-value	p-val vs.	p-value	Mean LS ±SEM	Max LS (75%)	worm no.
N2	wildtype	L4440	-	-	-			20.68±0.27	24	250
N2	wildtype	*bcat-1*	-	L4440	<0.0001			25.85±0.27	28	250
TU3311	*sid-1::gfp*	L4440	-	-	-			23.69±0.22	25	194
TU3311	*sid-1::gfp*	L4440/*let-363*	-	L4440	0.45			23.73±0.22	25	172
TU3311	*sid-1::gfp*	L4440/*bcat-1*	-	L4440	<0.0001	*let-363/ bcat-1*	<0.0001	26.24±0.18	28	292
TU3311	*sid-1::gfp*	*let-363/bcat-1*	-	*let-363*	<0.05			24.56±0.30	28	149
N2	wildtype	L4440	DMSO	-	-			23.00±0.24	25.2	247
N2	wildtype	*bcat-1*	DMSO	L4440	<0.0001	L4440/ rap	<0.0001	27.05±0.21	28.8	220
N2	wildtype	L4440	rapamycin [100 microM]	L4440	0.0148			23.94±0.29	26.4	216
N2	wildtype	*bcat-1*	rapamycin [100 microM]	*bcat-1*	<0.0001	L4440/rap	0.0705	24.85±0.23	27.2	232
CX3596	*(str-3::gfp)*	L4440	ablated ASI neuron	-	-			23.92±0.85	26	39
CX3596	*(str-3::gfp)*	*bcat-1*	ablated ASI neuron	L4440	0.734			24.46±0.94	28	37
CX3596	*(str-3::gfp)*	L4440	sham treated	-	-			20.42±0.31	22.8	133
CX3596	*(str-3::gfp)*	*bcat-1*	sham treated	L4440	<0.0001			23.75±0.45	26.8	89
PS3551	*hsf-1(sy441)*	L4440	-	-	-			18.94±0.26	20.8	250
PS3551	*hsf-1(sy441)*	*bcat-1*	-	L4440	0.671			18.84±0.28	20.4	245
EU31	*skn-1(zu135)*	L4440	-	-	-			18.39±0.19	20.2	250
EU31	*skn-1(zu135)*	*bcat-1*	-	L4440	<0.0001			22.47±0.24	25	221
CF1038	*daf-16(mu86)*	L4440	-	-	-			20.84±0.21	23.4	214
CF1038	*daf-16(mu86)*	*bcat-1*	-	L4440	0.0003			21.91±0.25	24.4	208
DR62	*daf-7(m62)*	L4440	5-FU	-	-			31.6±0.31	34.25	222
DR62	*daf-7(m62)*	*bcat-1*	5-FU	L4440	0.148			30.73±0.31	33.75	249

Supplementary Table 4 continued

strain	genotype	RNAi	treatment	p-val vs.	p-value	p-val vs.	p-value	Mean LS ±SEM	Max LS (75%)	worm no.
DR40	*daf-1(m40)*	L4440	5-FU	-	-			33.69±0.24	36	260
DR40	*daf-1(m40)*	*bcat-1*	5-FU	L4440	0.8383			33.23±0.28	36.6	251
N2	wildtype	L4440	-	-	-			20.59±0.20	22.2	313
N2	wildtype	L4440/ *daf-4*	-	L4440	<0.0001			25.06±0.25	28.2	280
N2	wildtype	L4440/ *bcat-1*	-	L4440	<0.0001			25.34±0.28	29	264
N2	wildtype	*daf-4/ bcat-1*	-	L4440	<0.0001	bcat-1	0.7953	25.37±0.24	27.8	269
N2	wildtype	-	H2O	-	-			25.30±0.21	27.3	292
N2	wildtype	-	L-Leucine [5mM]	H2O	<0.0001			27.03±0.29	29.3	306
N2	wildtype	-	L-Alanine[5mM]	H2O	0.243			25.69±0.25	27	228
CX3596	*(str-3::gfp)*	-	ablated ASI neuron +H2O	-	-			27.15±0.75	29.7	40
CX3596	*(str-3::gfp)*	-	ablated ASI neuron + L-leu [5mM]	H2O	0.9577			27.12±0.65	30.3	51
CX3596	*(str-3::gfp)*	-	sham treated + H2O	-	-			25.69±0.21	29	412
CX3596	*(str-3::gfp)*	-	sham treated +L-leu [5mM]	H2O	<0.001			28.76±0.25	32	406
PS3551	*hsf-1(sy441)*	-	H2O	-	-			25.76± 0.21	29	405
PS3551	*hsf-1(sy441)*	-	L-Leucine [5mM]	H2O	0.059			26.61±0.23	30	694
EU31	*skn-1(zu135)*	-	H2O	-				29.90±0.26	33	317
EU31	*skn-1(zu135)*	-	L-leucine [5mM]	H2O	0.0269			31.57±0.29	34	240
CF1038	*daf-16(mu86)*	-	H2O	-				21.24±0.17	23	462
CF1038	*daf-16(mu86)*	-	L-leucine [5mM]	H2O	0.2539			21.69±0.16	23.7	416
N2	*wildtype*	-	-	-	-			22.2±0.3	25	265
MIR23	*bcat-1::gfp*	-	-	wildtype	<0.001			19.66±0.29	22	262
N2	*wildtype*	L4440	-	-	-			21.46±0.17	24	360
N2	*wildtype*	L4440/ *bcat-1*	-	L4440	<0.0001	bcat-1/ hlh-15	0.0798	24.52±0.24	28	374
N2	*wildtype*	L4440/ *hlh-15*	-	L4440	<0.0001			24.43±0.29	28	299
N2	*wildtype*	bcat-1/ *hlh-15*	-	L4440	<0.0001			23.84±0.27	28	297
N2	*wildtype*	-	H2O	-	-			27.37±0.23	31	372
N2	*wildtype*	-	L-valine	H2O	<0.01			28.59±0.19	31	421
N2	*wildtype*	-	L-isoleucine	H2O	<0.0001			29.97±0.18	33	421

Supplementary Table 5: Putative transcription factor binding sites within 1 kb of the *bcat-1* promoter

TF name	start	end	sequence	p value
hlh-15	-473	-458	TCCCCACGTGCGCCCG	8.7e-07
mdl-1	-474	-459	CTCCCCACGTGCGCCC	3.3e-06
eor-1	-347	-333	CGAGAAACATAGAGA	1.3e-05
mxl-3	-474	-459	CTCCCCACGTGCGCCC	2.3e-05
hlh-15	-187	-172	ATAGCAGCTGGACATT	3.5e-05
hlh-10	-189	-173	GCATAGCAGCTGGACAT	3.6e-05
gei-11	-574	-563	CGGTCGGTCGCC	4.3e-05
skn-1	-848	-834	GAAATGATGAAGTAG	4.7e-05
hlh-4	-188	-172	CATAGCAGCTGGACATT	7.0e-05
efl-1	-231	-217	ATATGGCGCCAGTTC	7.3e-05
hlh-19	-188	-173	CATAGCAGCTGGACAT	7.5e-05
tra-1	-592	-585	CGGGAGGC	7.6e-05
hlh-15	-457	-442	CCACCACGGGGGCCCA	8.8e-05
ref-1	-475	-459	TCTCCCCACGTGCGCCC	9.7e-05

Supplementary Table 6: Sequences of PCR primers (cloning)

Primer sequences containing SmaI recognition site used for RNAi plasmid cloning (5'→3'):		
**Gene**	**Forward primer sequence**	**Reverse primer sequence**
act-4	TCCCCCGGGGGATTGGACGCCCAAGACATCAA	TCCCCCGGGGGATTTGAGCGTTGTGGGTGGAA
ssq-3	TCCCCCGGGAGCTGGAGGGTCTTCGACT	TCCCCCGGGACCTCCCATAGCTCCAGATCC
ifc-1	TCCCCCGGGCGCTGAGATCGCGTTGAAAA	TCCCCCGGGGTTGCTTGCTTTGCGTCCA
mrp-2	TCCCCCGGGGGACACAGATCCAACCGCTTCCA	TCCCCCGGGGGACACTCTTGTTGCGCACACTC
cft-1	TCCCCCGGGCATACGAGCTCGGCAAATGG	TCCCCCGGGAAAATTCAGTGTGGTTTCCCTCA
csp-2	TCCCCCGGGGGAACGTCTTGCTGGAGACATCC	TCCCCCGGGGGACTTACGCTCTTCTGCCCACT
fkh-7	TCCCCCGGGGGAGGGGACTGACGTATCGGTTG	TCCCCCGGGGGAGTCCATTGTGTGGCTCGCTA
M116.2	TCCCCCGGGGGATGAATGGCCGGATATGCTCG	TCCCCCGGGGGAGACTTCCGAGCACCGATGAA
Y71G21B.31	TCCCCCGGGGGACGCACCCCTTGATAAAGCCT	TCCCCCGGGGGATACTTGAACGCACCCACCTG

Supplementary Table 7: Sequences of PCR primers (qPCR)

Primer sequences used for quantitative PCR (5'→3'):		
**Gene**	**Forward primer sequence**	**Reverse primer sequence**
C. elegans		
bcat-1	GATTGGGATGCCGAGAGAGG	TTCTGGACGGAACATGCGAA
cdc-42	AGCCATTCTGGCCGCTCTCG	GCAACCGCTTCTCGTTTGGC
pmp-3	TGGCCGGATGATGGTGTCGC	ACGAACAATGCCAAAGGCCAGC
D. rerio		
bcat1	CCAGGCTGTTCTGCCCTG	CCGATGAGCAGACTTCAACA
tbp	CACTTCGAGCCAGAAATGCT	GCCAATCGAGACTGTTCCTC
insra	TCACCTGTGAGGTGTTCGAG	CTTCCTGGATACGCAGTGGT
M. musculus		
bcat1	GAAGGAGATGTTCGGCTCAG	TCAGTCAGCTTTCCCAGGAT
hmbs	GCGTTTTCTAGCTCCTTGGTAA	GAAATCATTGCTATGTCCACCA
gapdh	CAACAGCAACTCCCACTCTTC	GGTCCAGGGTTTCTTACTCCTT
